# Cephalopod-inspired optical engineering of human cells

**DOI:** 10.1038/s41467-020-16151-6

**Published:** 2020-06-02

**Authors:** Atrouli Chatterjee, Juana Alejandra Cerna Sanchez, Toyohiko Yamauchi, Vanessa Taupin, Justin Couvrette, Alon A. Gorodetsky

**Affiliations:** 1 Department of Chemical and Biomolecular Engineering, University of California, Irvine, Irvine, CA 92697 USA; 2 Department of Molecular Biology and Biochemistry, University of California, Irvine, Irvine, CA 92697 USA; 3 Beckman Laser Institute and Medical Clinic, University of California, Irvine, Irvine, CA 92697 USA; 4 Central Research Laboratory, Hamamatsu Photonics, Hamamatsu, Shizuoka 4348601 Japan; 5 Electron Microscopy Core, University of California, San Diego, La Jolla, CA 92093 USA; 6 Department of Materials Science and Engineering, University of California, Irvine, Irvine, CA 92697 USA; 7 Department of Chemistry, University of California, Irvine, Irvine, CA 92697 USA

**Keywords:** Genetic engineering, Synthetic biology, Optics and photonics

## Abstract

Although many animals have evolved intrinsic transparency for the purpose of concealment, the development of dynamic, that is, controllable and reversible, transparency for living human cells and tissues has remained elusive to date. Here, by drawing inspiration from the structures and functionalities of adaptive cephalopod skin cells, we design and engineer human cells that contain reconfigurable protein-based photonic architectures and, as a result, possess tunable transparency-changing and light-scattering capabilities. Our findings may lead to the development of unique biophotonic tools for applications in materials science and bioengineering and may also facilitate an improved understanding of a wide range of biological systems.

## Abstract (web summary)

While organisms like squid can adaptively modulate the optical properties of their tissues, human cells lack analogous abilities. Here the authors engineer human cells to produce protein architectures with tunable light scattering functionalities.

## Introduction

The idea of humans vanishing from sight by becoming transparent or invisible has captivated the imaginations of the general populace and scientists alike for millennia. These concepts have been described in classic literature by various authors, including the philosopher Plato, who conceived the Ring of Gyges as a hypothetical item that would allow its wearer to disappear^1^, and the writer H. G. Wells, who envisioned that a scientist could match their refractive index to that of air in order to become invisible^2^. Although such notions may seem fantastic at first glance, the natural world is filled with examples of animals, such as the glasswing butterfly^3^, the grass shrimp^4^, the comb jellyfish^5^, the glass frog^6^, and mesopelagic cephalopods^7^, which have evolved transparent structures, tissues, or even whole bodies for the purpose of concealment^8,9^. From a technological perspective, the study of transparency (defined as the property of transmitting light without appreciable scattering such that objects lying beyond can be seen clearly) has recently attracted significant attention. This renewed interest in transparency has been motivated by  the emergence of laboratory techniques for making deceased mammalian tissues/organs optically clear and thus amenable to three-dimensional visualization^10,11^. In this regard, whether in nature or in the laboratory, static transparency for biological systems has been typically achieved in the same way—by maximizing the direct transmission of visible light while simultaneously minimizing competing processes, i.e. the absorption of light by biomolecules found in the system of interest and the scattering of light due to differences in refractive index along its path^8–11^. However, technologically valuable methodologies for dynamically manipulating the transparency of living human cells and tissues have proven challenging to develop and effectively remain confined to the realm of science fiction.

**Fig. 1:**
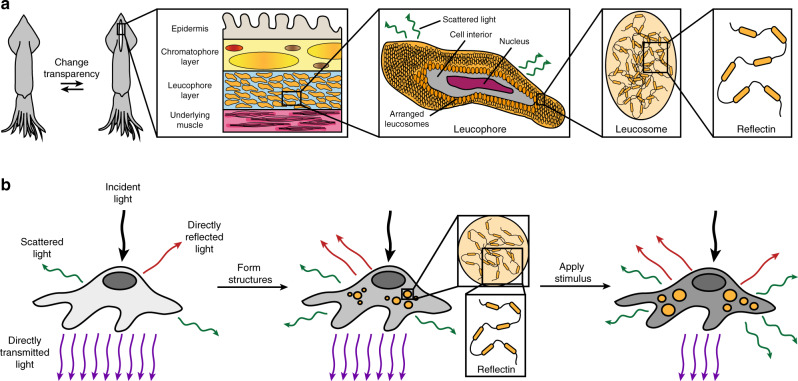
Overview of the biological inspiration and the design of human cells with tunable optical properties. **a** An illustration of a female *Doryteuthis opalescens* squid that switches a white stripe on its mantle from nearly transparent (left) to opaque white (right). (Inset, left) An illustration of a cross-section of the white stripe that shows the epidermis, chromatophore layer, leucophore layer, and underlying muscle. (Inset, middle left) An illustration of a leucophore, wherein the membrane contains an embedded arrangement of proteinaceous structures called leucosomes. The arrangement enables the cell to diffusely reflect, i.e. scatter, visible light. (Inset, middle right) An illustration of a leucosome, which contains assembled reflectin proteins. (Inset, right) A generalized illustration of a reflectin isoform. **b** (Left) A schematic of a human cell before transfection, which contains organelles as its only subcellular structures. The cell directly transmits (purple arrows) most of the incident visible light (black arrow) with relatively minimal scattering (green arrows). (Middle) A schematic of a human cell after the expression of reflectin and the formation of photonic architectures, i.e. a disordered arrangement of high refractive index, reflectin-based structures (orange circles), within its interior. The cell diffusely transmits and/or diffusely reflects, i.e. scatters (green arrows), some of the incident visible light (black arrow). (Right) A schematic of a human cell after exposure to a chemical stimulus that influences reflectin assembly, which demonstrates a plausible modification of the geometries and/or arrangements of its photonic architectures (orange circles). The cell now diffusely transmits and/or diffusely reflects, i.e. scatters (green arrows), a different amount of the incident visible light (black arrow).

In nature, adaptive transparency has been realized to some extent, with many cephalopods (i.e. octopuses, squids, and cuttlefish) demonstrating remarkable camouflage capabilities and even performing literal vanishing acts^12–17^. Indeed, these animals can dynamically alter how their skin transmits, absorbs, and reflects light through the functionality of unique natural optical components, which include pigmented organs called chromatophores, typically narrowband-reflecting cells called iridophores, and broadband-reflecting cells called leucophores^12–17^. As one specific example, the female *Doryteuthis opalescens* squid can avoid unwanted aggression by switching a stripe on its mantle from nearly transparent (i.e. weakly scattering) to opaque white (i.e. strongly scattering) (Fig. 1a and Supplementary Fig. 1)^17^. This feat represents a fascinating case study of adaptive biological optics and is thought to be achieved by means of a specialized layer that contains tunable leucophores (Fig. 1a and Supplementary Fig. 1)^17^. Generally, in octopus and cuttlefish skin, leucophores encompass disordered arrangements of proteinaceous structures called leucosomes, which range in diameter from hundreds of nanometers to several microns and can be membrane-bound or localized throughout the cells’ bodies (Supplementary Fig. 2)^18–20^. Such disordered leucosome arrangements (i.e. natural photonic architectures) allow cuttlefish leucophores to diffusely reflect (i.e. scatter) incident visible light via a Mie-type mechanism and to therefore function as passive broadband reflectors that produce bright white coloration^18–20^. In the female *D. opalescens* squid’s mantle, the leucophores contain similar leucosome arrangements (Fig. 1a and Supplementary Fig. 2), but rather than being passive, these cells are active, with broadband reflectances that can be reversibly modulated by injection of acetylcholine into the surrounding tissues (note that the exact molecular mechanisms underpinning such tunability are not yet fully understood) (Supplementary Fig. 1)^17^. Accordingly, dynamic cephalopod leucophores and their constituent light-reflecting photonic architectures constitute enticing archetypes for the design and engineering of other cellular systems with tunable optical properties.

Many of the internalized photonic architectures that enable the optical functionalities of cephalopod skin cells (including leucophores) are composed of proteins known as reflectins^13,21,22^. With a few exceptions, reflectins’ amino acid sequences consist of variable linker regions that are separated by conserved motifs with the highly general form (M/F-D-X_5_)(M-D-X_5_)_n_(M-D-X_3/4_)^13,21,22^. These sequences are unusual because they have a low percentage of common aliphatic amino acids, e.g. alanine, leucine, isoleucine, and a high percentage of aromatic amino acids, e.g. tyrosine and tryptophan, while also being enriched in arginine, asparagine, and methionine^13,21,22^. This peculiar composition is thought to be directly responsible both for reflectins’ remarkably diverse self-assembly properties^13,22^ and for their unusually high refractive indices^23,24^. In particular, reflectins not only form the spheroidal leucosomes found in leucophores^19,20^ and the membrane-enclosed platelets found in iridophores^25–27^ in vivo, but they also readily assemble into nanoparticles^24,28–30^, microfibers^24^, hexagonal plates^31^, and thin films^13,24,32–34^ in vitro. For some of these nano- and micro-structures, the application of different chemical stimuli can even modulate their aggregation state, e.g. NaCl and ionic strength for the nanoparticles^24,28^, or lead to disassembly/reassembly, e.g. aromatic compounds for the hexagonal plates^31^. Furthermore, reflectin-based structures have been proven to possess high refractive indices in varied contexts, with average values of ~1.44 reported for condensed platelets in squid iridophores^27^, ~1.51 observed for leucosomes in cuttlefish leucophores^19^, and ~1.54 to ~1.59 measured for reflectin-based films on solid substrates^24,32^. Overall, this combination of characteristics has made reflectins attractive brick and mortar materials for the design and construction of unique bioinspired optical systems.

Herein, we draw inspiration from female *D. opalescens* squids' leucophores and their constituent reflectin-containing leucosome arrangements for the development and engineering of human cells with tunable optical properties. First, we conceptualize human cells that would contain reconfigurable reflectin-based subcellular structures and, as a result, would possess stimuli-responsive transparency-changing capabilities. Next, we assemble and definitively characterize the desired proteinaceous photonic architectures within human cells. In turn, we evaluate the effect of such architectures on the optical characteristics and functionalities of the engineered cells. Last, we demonstrate that transmission and scattering of light by our cell cultures can be modulated with an external stimulus. Taken together, our findings may lead to the development of unique biophotonic technologies and thus afford exciting scientific opportunities across biology, materials science, and bioengineering.

## Results

### Human cells with designer optical properties

By drawing inspiration from cephalopod leucophores, we designed human cells that would contain stimuli-responsive photonic architectures and, as a consequence, would possess the ability to change their appearance and transmission of light. Towards this end, we first selected human embryonic kidney (HEK) 293 cells as the platform for integration of such architectures because these cells reliably express various recombinant proteins and can accumulate some foreign biomolecules within cytoplasmic inclusion bodies or phase-separated aggregates^35–37^. We in turn selected the reflectin A1 (RfA1) isoform as the constituent material for our architectures because its homologues feature refractive indices that are among the largest known for any protein^23,27,32^, assemble into a diverse array of ionic strength-responsive structures both in vitro and in squid skin cells^17,24–26,28^, and possess amino acid sequences that differ dramatically from those of mammalian proteins (Supplementary Fig. 3). Having made these selections, we assumed that, before transfection, the initially transparent native human cells would contain the usual mammalian organelles as their only subcellular structures and, therefore, would directly transmit most of the incident visible light with relatively minimal scattering (Fig. 1b, left). However, we expected that, after transfection with a vector encoding for RfA1, the human cells would express this reflectin isoform in reasonable quantities, assemble the foreign protein into leucosome-like cytoplasmic aggregates with unusually high local refractive indices, and then randomly distribute the aggregates in disordered arrangements throughout the cells’ interiors (Fig. 1b, middle). As such, we speculated that the RfA1-expressing cells would now contain reflectin-based photonic architectures with refractive indices that differ significantly from the surrounding cytoplasm and would consequently diffusely transmit and/or diffusely reflect (i.e. scatter) some of the incident visible light, thereby making the cells less transparent in analogy to passive cuttlefish leucophores (Fig. 1b, middle). Furthermore, we postulated that, upon exposure to chemical stimuli known to influence RfA1 assembly such as NaCl^24,28^, the RfA1-expressing cells would reconfigure the sizes, geometries, and/or arrangements of their internalized photonic architectures and would diffusely transmit and/or diffusely reflect (i.e. scatter) a different amount of the incident visible light, thereby altering the cells’ transparency in analogy to tunable squid leucophores (Fig. 1b, right). The overall approach would represent a powerful strategy for endowing living mammalian cells and tissues with tunable optical capabilities that emulate those reported for cephalopod skin components.

### Protein-based architectures in human cells

**Fig. 2:**
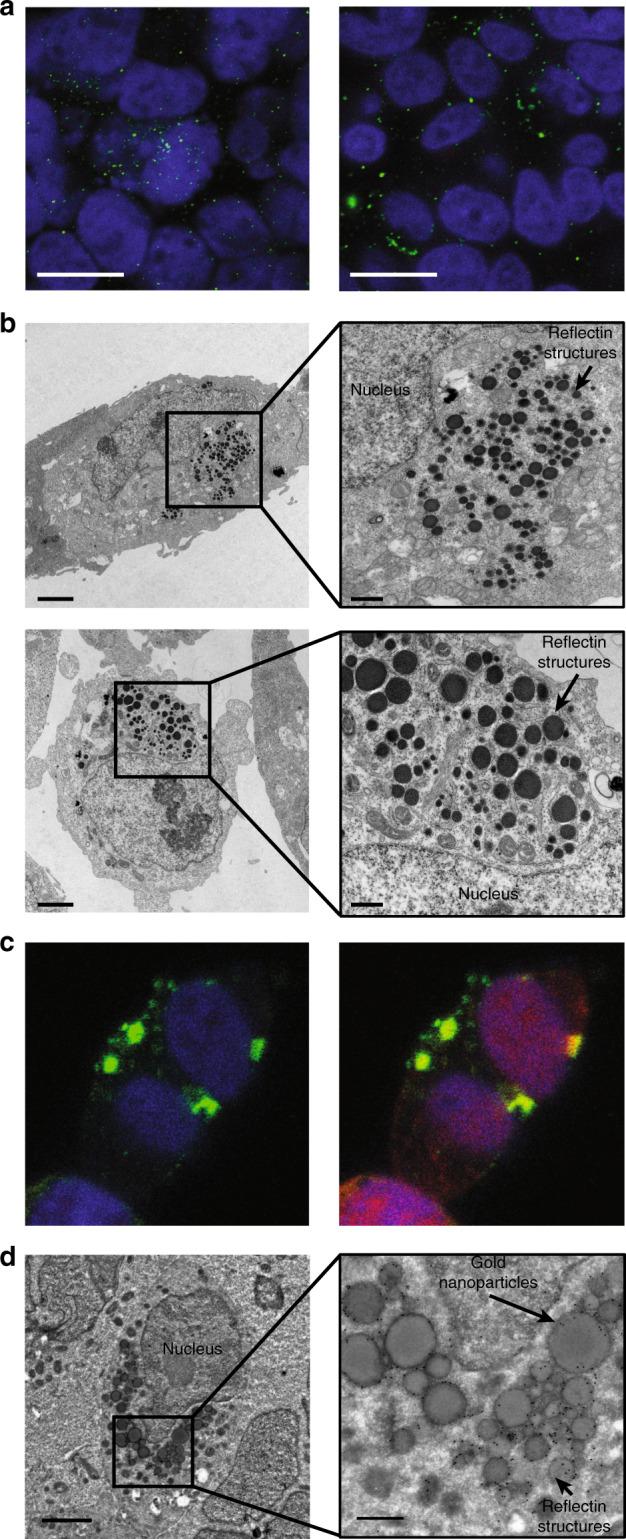
Confirmation of the formation of squid protein-based architectures in human cells. **a** (Left) Merged fluorescence microscopy images of fixed RfA1-expressing cells stained with DAPI and labeled with an antibody pair specific for the protein’s histidine-tag. (Right) Merged fluorescence microscopy images of fixed RfA1-expressing cells stained with DAPI and labeled with an antibody pair specific for reflectins’ unique sequence. The signals corresponding to DAPI and the Alexa 488 fluorophore-conjugated secondary antibody are colored blue and green, respectively, in both images. The scale bars are 15 µm in both images. **b** (Top) A TEM image of a cross-section from one representative RfA1-expressing cell, which reveals the presence of electron-dense structures. The scale bar is 2 µm. The inset shows a close-up image of a nanoparticle cluster. The scale bar is 500 nm for the inset. (Bottom) A TEM image of a cross-section from another representative RfA1-expressing cell, which also reveals the presence of electron-dense structures. The scale bar is 2 µm. The inset shows a close-up image of a nanoparticle cluster. The scale bar is 500 nm for the inset. **c** Merged fluorescence microscopy images of fixed RfA1- and RFP-expressing cells stained with DAPI and labeled with an antibody pair specific for reflectins’ unique sequence. (Left) The signals corresponding to DAPI and the Alexa 488 fluorophore-conjugated secondary antibody are shown, and they are colored blue and green, respectively. (Right) The signals corresponding to DAPI, the Alexa 488 fluorophore-conjugated secondary antibody, and RFP are shown, and they are colored blue, green, and red, respectively. The scale bars are 10 µm in both images. **d** (Left) An immuno-EM image of a cross-section from a representative cell, which has been labeled with a primary antibody specific for reflectins’ unique sequence followed by a complementary secondary antibody conjugated to a gold nanoparticle. The scale bar is 2 µm. (Right) A close-up image of a small cluster of RfA1 structures (large gray spheres), which are specifically labeled by antibody-conjugated gold nanoparticles (small black dots). The scale bar is 500 nm for the inset.

We began our studies by engineering human cells to produce large quantities of our squid protein. To this end, we grew HEK 293 cells transfected with a vector encoding for the expression of histidine-tagged *Doryteuthis pealeii* RfA1 and then visualized the fixed cells with immunofluorescence microscopy (see Methods for details). Here, the overlaid fluorescence microscopy images of fixed RfA1-transfected cells stained with the nuclear marker 4′,6′-diamidino-2-phenylindole (DAPI) and immunolabeled with an antibody pair specific for the proteins’s N-terminal histidine-tag revealed that the nuclei (colored blue) were surrounded by small reflectin aggregates (colored green), suggesting the successful expression of our squid protein by most of the cells (Fig. 2a, left). Similarly, the overlaid fluorescence microscopy images of fixed RfA1-transfected cells stained with DAPI, but now immunolabeled with an antibody pair specific for reflectins’ unique sequence, again, revealed that the nuclei (colored blue) were surrounded by reflectin aggregates (colored green), corroborating the successful expression of the squid protein by most of the cells (Fig. 2a, right). In comparison, the analogous fluorescence microscopy images of RfA1-transfected cells, for which immunolabeling was attempted with any member of the antibody pairs omitted, did not reveal any fluorescence signals (Supplementary Fig. 4). Furthermore, the fluorescence microscopy images of the mock transfected cells (i.e. ones treated with the transfection reagents but not the RfA1 vector) and the untransfected cells, for both of which immunolabeling was attempted with the reflectin-specific antibody pair, also did not reveal any fluorescence signals (Supplementary Figs. 5 and 6). These findings suggested that the transfected cells successfully expressed the non-native reflectin and then localized the protein within punctate aggregates.

We continued our studies by evaluating how the introduction of our squid protein affected the growth of the engineered human cells. Thus, we again cultured HEK 293 cells transfected with a vector encoding for the expression of histidine-tagged RfA1, initially visualized the live cells without any staining by using phase contrast microscopy, and then visualized both the live and fixed cells after staining with different markers by using fluorescence microscopy (see Methods for details). First, the phase contrast microscopy images of live RfA1-transfected cells indicated that the cells featured slightly rounded morphologies, whereas the analogous images of untransfected cells revealed typical spread-out morphologies (Supplementary Fig. 7). In addition, the fluorescence microscopy images of RfA1-transfected cells stained with both the live cell-specific calcein AM fluorescent dye and the dead cell-specific ethidium homodimer-1 fluorescent dye revealed a viability of 97 (±2)% and a density of 2.1 (±0.2) × 10^5^ cells per cm^2^, whereas the analogous images of live untransfected cells revealed an almost indistinguishable viability of 98 (±1)% and a slightly higher density of 2.8 (±0.4) × 10^5^ cells per cm^2^ (Supplementary Fig. 8). Moreover, the fluorescence microscopy images of fixed RfA1-transfected cells stained with fluorophore-tagged wheat germ agglutinin revealed areas of 338 (±50) µm^2^, whereas the analogous images of live untransfected cells revealed slightly larger areas of 375 (±50) µm^2^ (Supplementary Fig. 9). These experiments indicated that the expression of the non-native reflectin somewhat altered the engineered cells’ morphologies and areas but did not significantly impact their overall health.

Next, we proceeded to directly characterize the squid protein-based aggregates formed within our engineered human cells. For this purpose, we prepared thin cross-sections from fixed, resin-embedded, RfA1-expressing cells, and then imaged such cross-sections with transmission electron microscopy (TEM) (see the Methods for details). The TEM images obtained for multiple cellular cross-sections from RfA1-expressing cells revealed the presence of distinct arrangements of intracellular structures with large sizes and high electron densities, which presumably consisted of RfA1 and were found alongside the usual organelles, e.g. the nucleus, mitochondria, and ribosomes (Fig. 2b and Supplementary Fig. 10). These distinct arrangements routinely constituted >20% of the cellular cross-sections’ areas and generally consisted of two types of structures: (1) spheroidal nanoparticles with diameters of ~50 to ~250 nm typically located in clusters within the cells’ cytoplasm and (2) irregularly shaped nanostructures with diameters greater than ~250 nm often located closer to (or outside) the cells’ membranes and peripheries (Fig. 2b and Supplementary Figs. 10 and 11). In comparison, the analogous TEM images of cross-sections prepared either from cells that were not transfected with RfA1 or from cells that expressed red fluorescent protein (RFP), which can be aggregation-prone^38^, did not reveal structures resembling those found for the RfA1-expressing cells (Supplementary Figs. 12 and 13). Interestingly, the TEM images suggested that the engineered cells sequestered RfA1 within spheroidal nanoparticles, coalesced such nanoparticles into irregularly shaped nanostructures, and even expelled the larger structures into the surrounding environment. Together, these observations afforded detailed insight into the sizes, aggregation states, and subcellular distributions of the reflectin-based structures within our cells.

To better understand our squid protein’s subcellular localization, we investigated human cells that expressed not only histidine-tagged RfA1 but also red fluorescent protein (RFP) as a distinct biomolecular reporter. To this end, we grew HEK 293 cells transfected with a vector encoding for the expression of both histidine-tagged RfA1 and RFP as independent unconnected proteins, with the latter’s expression mediated by an internal ribosome entry site (IRES). We subsequently visualized such live cells with phase contrast and fluorescence microscopy and analogous fixed cells with immunofluorescence microscopy (see Methods for details). The overlaid phase contrast and fluorescence microscopy images of live RfA1- and RFP-transfected cells indicated that nearly two thirds of them expressed RfA1, as gauged from the fraction of the cell population that exhibited red fluorescence associated with the RFP reporter (Supplementary Fig. 14). The merged fluorescence microscopy images obtained for fixed RfA1- and RFP-expressing cells stained with DAPI and immunolabeled with an antibody pair specific for reflectins’ sequence revealed that the nuclei (colored blue) were in close proximity to RfA1 aggregates (colored green) (Fig. 2c), in agreement with the comparable images for the RfA1-expressing cells (Fig. 2a). The merged images obtained for RfA1- and RFP-expressing cells also revealed that the localized fluorescence from the immunolabeled RfA1-based structures (colored green) did not precisely overlap with the more dispersed fluorescence from the independent RFP reporter proteins (colored red), suggesting that the two biomolecules were distributed throughout the cells in different ways (Fig. 2c). In comparison, the analogous fluorescence microscopy images of fixed RfA1- and RFP-transfected cells, for which labeling was attempted with either the primary or secondary member of the reflectin-specific antibody pair omitted, only revealed fluorescence signals associated with RFP but not with specific immunolabeling (Supplementary Fig. 15). Overall, these findings further confirmed that our engineered human cells readily expressed reflectin in high yield and distributed this protein as variable-sized aggregates throughout the cells’ interiors in unique fashion.

We subsequently sought to unequivocally prove that the aggregates found within the engineered human cells were formed from our squid protein. For this purpose, we first prepared ultra-thin cross-sections from fixed, cryoprotectant-treated RfA1- and RFP-expressing cells; immunolabeled the sections via treatment with a primary antibody specific for reflectins’ sequence followed by a complementary secondary antibody conjugated to a gold nanoparticle; and then imaged the resulting labeled sections with electron microscopy (immuno-EM) (see the Methods for details). The representative immuno-EM images of such cellular sections revealed the presence of clusters of electron-dense structures (dark gray spheres) that were distributed throughout the cells’ interiors alongside the usual organelles, e.g. the nucleus (Fig. 2d). Such arrangements constituted a significant fraction of the cross-sections’ areas (and, presumably, of the cellular volumes) and typically consisted of spheroidal structures with diameters of tens to hundreds of nanometers (Fig. 2d). In general, the observed aggregates appeared similar in most respects, i.e. size, shape, location, and distribution, to those imaged with TEM not only in cells that expressed RfA1 (Fig. 2b and Supplementary Fig. 10) but also in cells that expressed both RfA1 and RFP (Supplementary Fig. 16). Most importantly, higher-magnification immuno-EM images of the cellular sections showed that our spheroidal structures were selectively labeled by the antibody-conjugated gold nanoparticles (small black dots), thus conclusively demonstrating that the structures consisted of RfA1 (Fig. 2d). Interestingly, the assorted high-resolution immuno-EM and TEM images obtained for the RfA1-based structures (Fig. 2b, d, and Supplementary Figs. 10 and 16) were reminiscent of classic and recent electron microscopy images of reflectin-based leucosomes from octopus, cuttlefish, and squid leucophores, although our structures’ size distributions differed somewhat from those reported for cuttlefish leucosomes (Supplementary Figs. 2 and 11)^17–20^. Together, these observations definitively and unambiguously confirmed the formation of unique arrangements of reflectin-based structures within our engineered human cells.

### Optical characteristics of the engineered cells

**Fig. 3:**
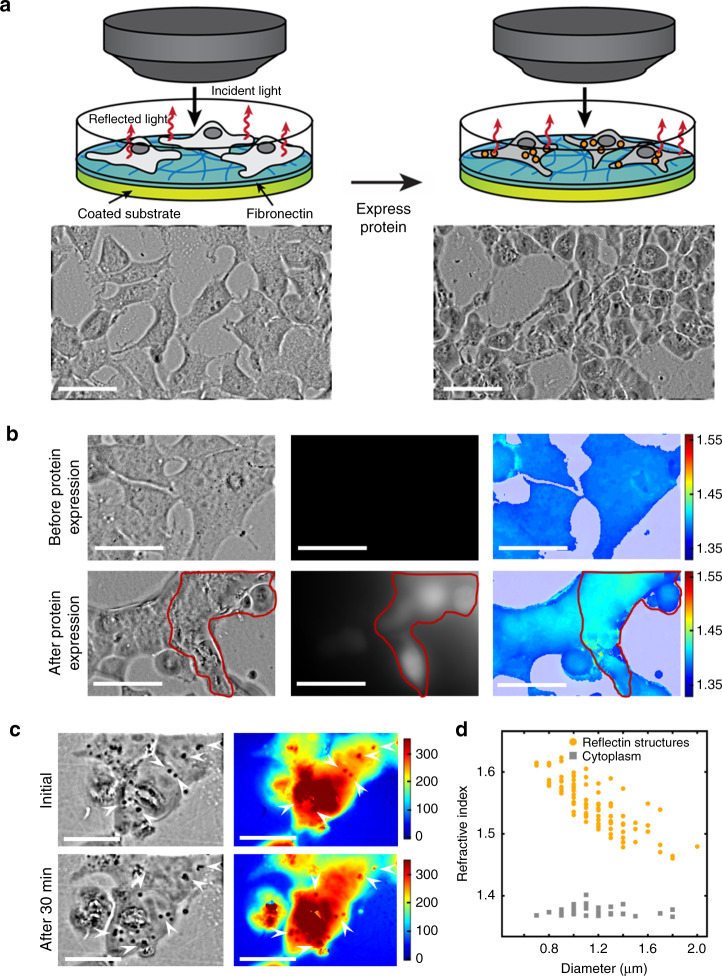
Evaluation of the optical characteristics of engineered squid protein-producing human cells. **a** (Left) A schematic (top) and reflection-mode quantitative phase microscopy image (bottom) of recently transfected cells. The cells possess moderate phase differences with the underlying substrate. (Right) A schematic (top) and reflection-mode quantitative phase microscopy image (bottom) of transfected cells that express RfA1. The cells possess more pronounced phase differences with the underlying substrate. The scale bar is 15 µm in both images. **b** (Top row) A reflection-mode quantitative phase microscopy image (left), fluorescence microscopy image (middle), and refractive index map (right) obtained for recently transfected cells, which do not yet express RfA1 and RFP. The cells exhibit no fluorescence and have a relatively low average refractive index. The scale bars are 15 µm in the images. (Bottom row) A reflection-mode quantitative phase microscopy image (left), fluorescence microscopy image (middle), and refractive index map (right) obtained for transfected cells, some of which express RfA1 and RFP. The cells that express the proteins (outlined in red) exhibit fluorescence signals and have a higher average refractive index. The scale bars are 15 µm in the images. **c** (Top row) A transmission-mode quantitative phase microscopy image (left) and corresponding optical pathlength map (right) obtained initially during the imaging of cells that express RfA1. The cells contain numerous RfA1-based structures with higher phases (dark black spots labeled with white arrows, left) and longer optical pathlengths (dark red spots labeled with white arrows, right) relative to their immediate surroundings. The scale bar is 10 µm in both images. (Bottom row) A transmission-mode quantitative phase microscopy image (left) and corresponding optical pathlength map (right) obtained 30 min later during the imaging of cells that express RfA1. The cells contain numerous RfA1-based structures which have changed their positions but still feature higher phases (dark black spots labeled with white arrows, left) and longer optical pathlengths (dark red spots labeled with white arrows, right) relative to the immediate surroundings. The scale bars are 10 µm in both images. **d** A plot of the calculated refractive index as a function of the diameter for readily-distinguished RfA1-based structures (orange dots) and for analogous cytoplasmic regions (gray dots) specifically found near the peripheries of cells that express RfA1.

We proceeded to study whether the expression of the squid protein modified our engineered human cell cultures’ interaction with light. Towards this end, we grew HEK 293 cells transfected with a vector encoding for the expression of histidine-tagged RfA1 and then visualized the resulting cell cultures with reflection-mode low coherence quantitative phase microscopy (RLC-QPM) (see Fig. 3a and the Methods for details). This technique measures how incident light changes its phase when reflected from various objects, such as living cells positioned on a substrate, and therefore enables the generation of phase images, which quantitatively represent the observed phase shifts (see Fig. 3a and the Methods for details)^39–41^. Initially, the RLC-QPM images obtained for recently transfected cells, which did not have enough time to express RfA1, showed that the cells had typical spread-out morphologies (Fig. 3a, left), in agreement with standard phase contrast microscopy imaging of untransfected cells (Supplementary Fig. 7). The phase difference between the cells’ bodies (relatively dark gray areas) and the glass substrate (relatively light gray areas) was moderate, although the bodies featured some regions with a greater phase difference (small gray-black spots) that likely corresponded to organelles (Fig. 3a, left), in agreement with TEM images of untransfected cells (Supplementary Fig. 12). In comparison, the RLC-QPM images obtained for analogous cells that had been given sufficient time to express RfA1 showed that they now had slightly rounded morphologies (Fig. 3a, right), in agreement with standard phase contrast microscopy imaging of RfA1-expressing cells (Supplementary Fig. 7). The phase difference between the cells’ bodies (now even darker gray areas) and the glass substrate (relatively light gray areas) had become more pronounced, with the cell bodies featuring a substantial number of regions with a higher phase difference (large dark black spots) that likely corresponded to RfA1-based nanostructures, in agreement with TEM images of RfA1-expressing cells (Fig. 2b and Supplementary Fig. 10). These experiments showed that the formation of disordered arrangements of reflectin-based structures within our cells’ interiors altered the way in which they reflected light.

Next, we sought to explicitly quantify the effect of the squid protein on the optical characteristics (specifically, the refractive indices) of our engineered human cells. Thus, we grew cells transfected with a vector encoding for the expression of both histidine-tagged RfA1 and the RFP reporter and, again, visualized the resulting cells with both RLC-QPM and fluorescence microscopy (on a single instrument). Here, RLC-QPM facilitated recording of not only the phase images but also the corresponding optical pathlengths and geometric heights for different cells before and after protein expression, subsequently enabling precise calculation and comparison of the cells’ refractive index maps (see the Methods for details)^39–41^. In tandem, fluorescence microscopy provided verification of RfA1 expression via monitoring of the RFP reporter, making it possible to unambiguously differentiate between the RfA1-expressing and the likely untransfected cells. At first, the RLC-QPM images of recently transfected cells, which were not yet expressing RfA1 and RFP, showed that the cells featured a relatively minimal phase difference with the substrate (Fig. 3b, top left). The fluorescence microscopy images of the same cells did not reveal the presence of any RFP-associated fluorescence signals (Fig. 3b, top middle). Furthermore, the corresponding cellular refractive index distribution was generally uniform with an average value of ~1.38 (Fig. 3b, top right), which matched literature precedent for whole mammalian cells^39,42,43^. Subsequently, the RLC-QPM images of cells that had been given sufficient time to express RfA1 and RFP showed that some of the cells now featured a more pronounced phase difference with the substrate, presumably due to the presence of RfA1-based structures (Fig. 3b, bottom left). The fluorescence microscopy images of the same cells revealed that some of the cells exhibited clear RFP-associated fluorescence signals, confirming successful protein expression (Fig. 3b, bottom middle). Furthermore, the corresponding cellular refractive index distributions were no longer uniform: the non-fluorescent cells, which had failed to express both RfA1 and RFP, retained an average refractive index of ~1.38, whereas the fluorescent cells, which had successfully expressed both proteins, now featured an average refractive index of >~1.42 (Fig. 3b, bottom right). Notably, for measurements performed across multiple cultures, the average refractive indices were consistently lower for the non-fluorescent (i.e. non-protein producing) cells and remained relatively unchanged for days, whereas the average refractive indices were consistently higher for the fluorescent (i.e. protein producing) cells and remained relatively unchanged only after the emergence of fluorescence (Supplementary Fig. 17). As might be expected, the RfA1- and RFP-expressing cells did exhibit some variability in their refractive index values, with more intense fluorescence signals and greater structure/aggregate volume fractions typically correlating to higher indices. Together, these measurements demonstrated that our cells’ refractive indices could be engineered through the introduction of arrangements of reflectin-based structures, i.e. photonic architectures, within their interiors.

In turn, we investigated the influence of the squid protein-based structures on the local propagation of light through our engineered human cells. Towards this end, we specifically probed RfA1-expressing HEK 293 cells containing multiple large, readily distinguished structures (or possibly nanoparticle clusters) with TLC-QPM in real time. This technique measures how incident light changes its phase when transmitted by different objects, such as living cells positioned on a substrate, and therefore enables generation of both phase images and corresponding optical pathlength maps (see the Methods for details)^39–41^. Initially, the TLC-QPM images obtained for RfA1-expressing cells revealed numerous higher-phase structures (dark black spots, white arrows), which were distributed throughout the cells’ interiors and possessed apparent diameters on the order of ~0.8 to ~2 μm (Fig. 3c, top left). The corresponding optical pathlength maps indicated that the longer pathlengths (with respect to the immediate surroundings) were exactly correlated to the structures’ subcellular locations (dark red spots, white arrows), suggesting that their presence had substantially modified the local refractive index (Fig. 3c, top right). Subsequently, the TLC-QPM images obtained for the same RfA1-expressing cells after a period of 30 min revealed that they still contained numerous higher-phase structures (dark black spots, white arrows), which had maintained their apparent sizes but changed their positions (Fig. 3c, bottom left). The corresponding optical pathlength maps indicated that the longer pathlengths (again with respect to the immediate surroundings) had analogously shifted but were still correlated to the structures’ subcellular locations (dark red spots, white arrows), suggesting a concomitant spatial redistribution of the modifications in the local refractive index (Fig. 3c, bottom right). Interestingly, the time-lapse videos generated from multiple phase images and optical pathlength maps, which were collected for the cells at different time intervals, demonstrated that the RfA1-based structures dynamically moved within the cytoplasm and occasionally congregated near the membranes, positioning themselves for release into the surroundings within extracellular vesicles (Supplementary Movies 1 and 2). The findings were in general agreement with our analyses of the TEM images obtained for the RfA1-expressing cells, which captured snapshots of the expulsion of the larger structures by the cells (Fig. 2b and Supplementary Fig. 10). These experiments demonstrated that the specific position or arrangement of the reflectin-based structures within our cells’ interiors determined the way in which they locally transmitted light.

Subsequently, we quantified and analyzed the optical characteristics of the individual squid protein-based structures within our engineered human cells. Specifically, we calculated the refractive indices of the readily distinguished structures, which appeared somewhat larger than those most frequently observed with TEM, positioned near the perimeters of the RfA1-expressing cells. For the purpose of these calculations, we assumed that the structures’ diameters, as roughly estimated from the TLC-QPM images, were similar to the cells’ heights at their peripheries, and used the equation ${\mathrm{OPL}} = \frac{{\lambda \Delta \phi }}{{4\pi }} = {\it{d}}\left( {{\it{n}}_{\mathrm{a}} - {\it{n}}_{\mathrm{s}}} \right)$, where OPL is the optical pathlength, *λ* is the central wavelength of the imaging light, Δ*ϕ* is the phase difference, *d* is the apparent diameter of the structure, *n*_a_ is the refractive index of the structure, and *n*_s_ is the refractive index of the immediate surroundings^39,40^. The calculations performed for an ensemble of representative RfA1-based structures yielded size-dependent refractive indices that varied from ~1.48 to ~1.62, with the higher and lower values generally corresponding to the smaller and larger apparent diameters, respectively (Fig. 3d). These values were comparable to the refractive indices of ~1.44 reported for reflectin-filled condensed lamella in iridophores^27^, ~1.51 measured for reflectin-containing leucosomes in leucophores^19^, and ~1.54 to ~1.59 found for substrate-bound reflectin films^24,32^. In contrast, the analogous calculations performed for representative regions of the cytoplasm proximal to the large structures yielded size-independent refractive indices that varied from ~1.36 to ~1.39 (Fig. 3d). These values were comparable to the refractive indices of ~1.35 to ~1.37 previously reported for the cytoplasm of mammalian cells^44–46^. Interestingly, the refractive index distribution for the larger RfA1-based nanostructures found at the perimeters of our engineered human cells (Fig. 3d) roughly resembled the refractive index distribution for the larger reflectin-containing leucosomes found inside cuttlefish leucophores (Supplementary Fig. 18)^19^. In their totality, our observations intimated that it might, in principle, be possible to more precisely engineer our cells’ optical characteristics by expressing reflectins conjugated with known targeting peptides and inducing such proteins to form well-defined distributions of high refractive index aggregates in specific subcellular locations.

### Tunable optical properties for the engineered cells

**Fig. 4:**
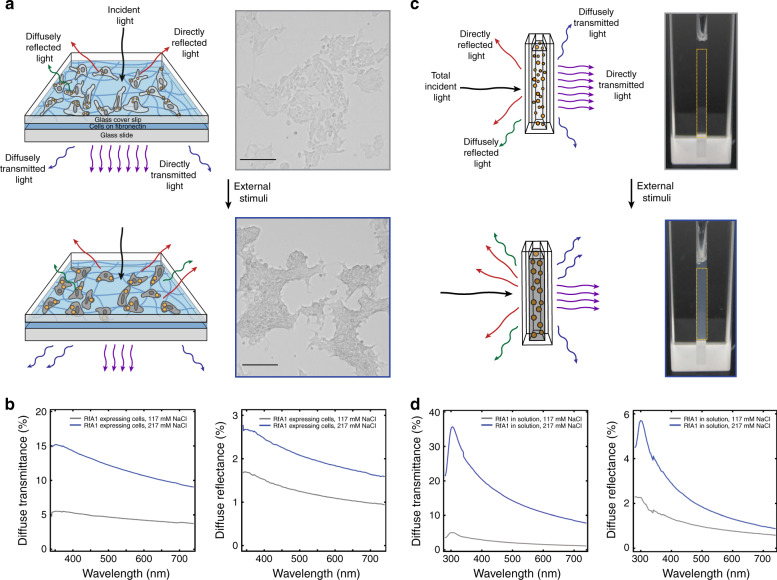
Demonstration of tunable optical properties for engineered human cell cultures. **a** (Top) A schematic (left) and representative brightfield microscopy image (right) of a sandwich-type configuration, wherein the middle layer consists of an RfA1-expressing cell culture, after exposure to media with a standard, i.e. 117 mM, NaCl concentration. The cells do not substantially attenuate incident light and appear similar to their environment. (Bottom) A schematic (left) and representative brightfield microscopy image (right) of a sandwich-type configuration, wherein the middle layer consists of an RfA1-expressing cell culture, after exposure to media with a higher, i.e. 217 mM, NaCl concentration. The cells more strongly attenuate light and appear relatively distinct from their environment. The scale bars are 225 µm in both images. **b** (Left) Representative diffuse transmittance spectra obtained for sandwich-type configurations from RfA1-expressing cells after exposure to media with standard (gray trace) and higher (blue trace) NaCl concentrations. (Right) Representative diffuse reflectance spectra obtained for sandwich-type configurations from RfA1-expressing cells after exposure to media with standard (gray trace) and higher (blue trace) NaCl concentrations. **c** (Top) A schematic (left) and representative digital camera image (right) of a cuvette containing an aqueous RfA1 solution with a 117 mM NaCl concentration. The solution, which contains small RfA1 nanoparticles (orange spheres), appears visibly transparent and weakly scatters incident light. (Bottom) A schematic (left) and representative digital camera image (right) of a cuvette containing an aqueous RfA1 solution with a 217 mM NaCl concentration. The solution, which contains much larger RfA1 nanoparticles (orange spheres), appears visibly opaque and more strongly scatters incident light. **d** (Left) Representative diffuse transmittance spectra obtained for aqueous RfA1 solutions with 117 mM (gray trace) and 217 mM (blue trace) NaCl concentrations. (Right) Representative diffuse reflectance spectra obtained for aqueous RfA1 solutions with 117 mM (gray trace) and 217 mM (blue trace) NaCl concentrations.

Last, we sought to assess whether the light-transmitting properties of our engineered human cell cultures could be controllably modulated, i.e. tuned, with an external chemical stimulus. To accomplish this goal, we designed and prepared sandwich-type configurations, wherein the bottom layer was a fibronectin-coated glass slide, the middle layer was fixed RfA1-expressing HEK 293 cells exposed to media with different ionic strengths (i.e. distinct NaCl concentrations), and the top layer was a glass coverslip overlaid onto the cells (see Fig. 4a and the Methods for details). We then visualized the cell cultures with brightfield microscopy, a technique which measures how incident light is attenuated upon transmission through different objects and furnishes corresponding brightness/intensity images that are readily analyzed via digital image processing methods^47,48^. Specifically, for RfA1-expressing cell cultures exposed to media with a standard (i.e. 117 mM) NaCl concentration, the representative brightfield microscopy images obtained revealed that the cells did not substantially attenuate the incident light and appeared similar to the environment (Fig. 4a, bottom). For RfA1-expressing cell cultures exposed to media with a higher (i.e. 217 mM) NaCl concentration, the analogous representative images revealed that the cells now more strongly attenuated the incident light and appeared relatively distinct from the environment (Fig. 4a, bottom). Additionally, for RfA1-expressing cells exposed to the standard NaCl concentration media, the brightfield microscopy images’ representative histograms of the number of pixels at different intensity values spanned a relatively narrow range of ~175 to ~215 with a maximum at ~195, thus quantitatively confirming the cells’ similarity to the surroundings (Supplementary Fig. 19a). For RfA1-expressing cells exposed to the higher NaCl concentration media, the analogous representative histograms spanned a wider range of ~130 to ~220 with a shifted maximum at ~187, thus quantitatively confirming the cells’ increased contrast with the surroundings (Supplementary Fig. 19b). By comparison, the representative brightfield microscopy images obtained for untransfected cell cultures revealed that they attenuated less incident light than the RfA1-expressing cells and did not substantially change appearance after exposure to the higher NaCl concentration media (Supplementary Fig. 20). The images’ representative histograms for the untransfected cells were comparable to those of the cells’ surroundings and also remained relatively unchanged after exposure to the higher NaCl concentration media (Supplementary Fig. 21). Notably, the fluorescence microscopy images of live RfA1-expressing and untransfected cells stained with the calcein AM and ethidium homodimer-1 dyes showed that their viabilities and densities were unaffected by the media’s NaCl concentration (Supplementary Fig. 22). The fluorescence microscopy images of fixed RfA1-expressing and untransfected cells stained with fluorophore-tagged wheat germ agglutinin likewise showed that their areas were unaffected by the media’s NaCl concentration (Supplementary Fig. 23). Together, these experiments suggested, yet again, that our engineered cells’ internalized, high refractive index, reflectin-based photonic architectures determined the way in which they transmitted light and also showed that exposure of such cells to variable ionic strength media did not influence their viabilities or morphologies but did alter (tune) their light-transmitting properties (i.e. transparency with respect to the surroundings).

Next, we investigated precisely why the engineered human cell cultures’ transmission and reflection of light were affected by the application of a chemical stimulus. For this purpose, we again prepared sandwich-type configurations from RfA1-expressing cells that had been exposed to media with different ionic strengths (i.e. distinct NaCl concentrations) and then systematically characterized these configurations with a combination of transmittance and reflectance spectroscopy (see the Methods for details). First, the representative total transmittance and total reflectance spectra recorded for the RfA1-expressing cell cultures indicated that their broadband transmittance decreased and that their broadband reflectance increased after exposure to the higher NaCl concentration media (Supplementary Fig. 24). In comparison, the representative total transmittance and total reflectance spectra recorded for the untransfected cell cultures indicated that their broadband transmittances and reflectances were larger and smaller, respectively, than those of the RfA1-expressing cell cultures and were relatively unchanged by exposure to the higher NaCl concentration media (Supplementary Fig. 25). In addition, the diffuse transmittance and diffuse reflectance spectra recorded for the RfA1-expressing cell cultures revealed that the diffuse components of the transmittance increased from ~5.5 (±1.5)% to ~13 (±2.1)% (i.e. by >~2-fold) and that the diffuse components of the reflectance increased from ~1.1 (±0.1)% to ~2.1 (±0.2)% (i.e. by ~2-fold) after exposure to the higher NaCl concentration media (Fig. 4b and Supplementary Fig. 26). In comparison, the diffuse transmittance and diffuse reflectance spectra recorded for the untransfected cell cultures revealed that the diffuse reflectance and transmittance components were both lower than those of the RfA1-expressing cell cultures and also were relatively unchanged by exposure to the higher NaCl concentration media (Supplementary Figs. 26 and 27). Furthermore, the transmittance and reflectance spectra obtained for fibronectin matrices in the same configuration but without any cells revealed relatively high total transmittances and low total reflectances with minor background diffuse components (Supplementary Figs. 25 and 27). Interestingly, the NaCl concentration-induced tuning of the transparency and broadband diffuse reflectance for our sandwich-type configurations from RfA1-expressing cells (Fig. 4a, b) bore a superficial resemblance to the acetylcholine-triggered switching of the opacity and broadband reflectance for female *D. opalescens* squids’ leucophore-containing layers (Supplementary Fig. 1). Overall, the spectroscopic measurements demonstrated that the introduction of internalized, high refractive index, reflectin-based photonic architectures caused our cells to diffusely transmit and/or diffusely reflect (i.e. scatter) more of the incident visible light and that exposure of these engineered cells to variable ionic strength media further enhanced (tuned) their scattering of visible light, thus providing an explanation for their ability to change transparency with respect to the surroundings.

To better understand the mechanistic underpinnings of our engineered human cells’ tunable scattering of incident light, we employed aqueous reflectin solutions as in vitro model systems and investigated how the solutions’ appearances, transmittances, and reflectances were influenced by our chosen chemical stimulus. For this purpose, we first solubilized histidine-tagged RfA1 that had been heterologously expressed in *E. coli* and then systematically increased the ionic strength (i.e. NaCl concentration) of the resulting aqueous solutions, while characterizing them with a synergistic combination of digital camera imaging, transmittance and reflectance spectroscopy, and dynamic light scattering (DLS) (see the Methods for details). First, the representative digital camera images, total transmittance spectra, and total reflectance spectra obtained for RfA1 solutions with a standard (i.e. 117 mM) NaCl concentration revealed that they were visibly transparent to the naked eye, transmitted most of the incident light, and reflected little of the incident light, respectively (Fig. 4c and Supplementary Fig. 28). In contrast, the representative digital camera images, total transmittance spectra, and total reflectance spectra obtained for RfA1 solutions with a high (i.e. 217 mM) NaCl concentration revealed that they were visibly opaque to the naked eye, transmitted much less of the incident light, and reflected slightly more of the incident light, respectively (Fig. 4c and Supplementary Fig. 28). In addition, the DLS measurements, diffuse transmittance spectra, and diffuse reflectance spectra obtained for the solutions with the standard NaCl concentration revealed that they primarily contained RfA1 nanoparticles with diameters of ~36 (±10) nm and featured relatively low diffuse transmittance and diffuse reflectance components with average values of ~3.3 (±1.0)% and ~0.8 (±0.2)%, respectively, indicating that such solutions very weakly scattered visible light (Fig. 4d and Supplementary Figs. 29 and 30). In comparison, the DLS measurements, diffuse transmittance spectra, and diffuse reflectance spectra obtained for the solutions with the higher NaCl concentration revealed that they primarily contained RfA1 nanoparticles with diameters of ~106 (±9) nm and featured much larger diffuse transmittance and diffuse reflectance components with average values of ~15.1 (±4.4)% and ~1.9 (±0.3)%, respectively, indicating that such solutions more strongly scattered visible light presumably via a Mie-type mechanism (Fig. 4d and Supplementary Figs. 29 and 30)^49^. In general, for our RfA1 solutions, the diameters of their constituent nanoparticle populations and the values of their diffuse transmittance and diffuse reflectance components increased as a function of the NaCl concentration, underscoring the fact that the sizes and/or aggregation states of the RfA1-based nanoparticles determined the solutions’ associated degree of light scattering and thus overall transparencies (Supplementary Figs. 29 and 30)^49^. Moreover, the analogous aqueous solutions without RfA1 nanoparticles featured relatively low background diffuse transmittances and diffuse reflectances and were completely transparent to the naked eye, further supporting our analysis (Supplementary Fig. 30). The in vitro measurements suggested that external ionic strength changes could reconfigure the sizes, geometries, and/or arrangements of our engineered cells’ internalized, high refractive index, reflectin-based photonic architectures and consequently could modulate the cells’ diffuse transmission and/or reflection, i.e. scattering, of incident visible light. As such, when considered within the context of the in vivo observations presented above and the previous findings for light scattering by leucosome arrangements in leucophores^17–20^, our experiments together provided a plausible explanation for the origins of our engineered cellular systems’ tunable optical properties.

## Discussion

In summary, by drawing inspiration from the subcellular structures and adaptive optical functionalities of cephalopod leucophores, we have conceptualized and realized human cells that encompass reconfigurable protein-based photonic architectures and thus possess tunable light-transmitting and light-reflecting capabilities. We believe that our findings hold broad scientific and technological significance for multiple reasons. First, our work introduces the concept of directly engineering the optical properties, i.e. refractive index, and extent of light scattering, for living human cells via the incorporation of reflectin-based structures and, therefore, lays the groundwork for the development of mammalian cells and organoids with other sophisticated cephalopod-inspired optical functionalities, such as stimuli-responsive dynamic iridescence or mechanically reconfigurable coloration^26,50^. Second, the unexpected observation that reflectin-based structure arrangements not only readily self-assemble but also maintain their high refractive indices within the foreign biological environment of human cells suggests that common paradigms, e.g. sequence motifs, may underpin the structures and functions of reflectin-based architectures within the proteins’ diverse native biological environments, which include chromatophore sheath cells^50^, iridophores^25–28^, and leucophores^17–20^. Third, given that such native cephalopod skin cells remain quite challenging to culture, the reported designer mammalian cells may constitute appropriate surrogate model frameworks for making further discoveries with regard to the properties of reflectins and the molecular and cellular biology of molluscs. Fourth, reflectins’ unique orthogonal amino acid sequences and validated high refractive indices in vivo could make them ideal biomolecular reporters for the quantitative phase microscopy visualization of varied cellular processes, e.g. expulsion of extracellular vesicles, across typically non-transparent biological specimens^41^, in approaches reminiscent of the ones pioneered for jellyfish green fluorescent proteins with fluorescence microscopy^51,52^. Fifth, reflectins’ diverse stimuli-responsive self-assembly properties and ease of expression reported herein may enable real-time adaptive refractive index matching of specific mammalian cells to their surroundings and, thus, facilitate imaging of entire living tissues with improved clarity and resolution on conventional optical microscopes, as done in studies of static deceased tissues with seminal clearing techniques^10,11^. In general, the continued development and exploration of engineered reflectin-producing systems via the aforementioned strategies could help answer fundamental questions associated with three-dimensional inter- and intra-cellular organization relevant for light-cell and light-tissue interactions in both cephalopods and other animals. Consequently, our findings may afford a variety of exciting opportunities and possibilities within the fields of biology, materials science, and bioengineering.

## Methods

### Growth and transfection of human cells

The human embryonic kidney (HEK) 293 cells (ATCC, CRL-1573^TM^) were grown and transfected according to standard protocols. First, vector constructs encoding for the independent expression of N-terminal histidine-tagged *D. (Loligo) pealeii* reflectin A1 (RfA1) (Genbank: ACZ57764.1), the independent expression of Cayenne Red Fluorescent Protein (RFP), or the expression of both RfA1 and RFP (with the expression of the latter mediated by an internal ribosome entry site from the encephalomyocarditis virus) were designed by ATUM using their Gene Designer Software. The vectors all contained 5′UTR regions downstream of a cytomegalovirus promoter and enhancer, a standard origin of replication derived from pBR322, a polyadenylation signal to aid in the termination of transcription, and cDNA encoding for the protein or proteins of interest. Subsequently, HEK 293 cells (ATCC, CRL-1573^TM^) were cultured on plastic or fibronectin-coated glass dishes in Minimal Essential Medium (MEM) supplemented with Earle’s salts and 10% fetal bovine serum (FBS) (Life Technologies) at a temperature of 37 °C and under 5% CO_2_. For transfection, the HEK 293 cells were seeded at ~5% to ~33% of the confluent density for the plastic or glass dishes and grown for another ~14 to ~24 h. The medium was swapped for MEM supplemented with Earle’s salts but lacking FBS. A transfection reagent mixture containing Lipofectamine 2000 (Life Technologies) and a vector encoding for just RfA1, just RFP, or both RFA1 and RFP (ATUM) was added to the medium, and the cells were incubated for ~24 to ~48 h. Typically, the untransfected or transfected cell cultures were grown to confluencies of ~50% to ~75%. The cells were fixed as necessary, used for the preparation of cellular cross-sections, or directly characterized with microscopy techniques.

### Expression and purification of reflectin A1 in bacteria

N-terminal histidine-tagged RfA1 was expressed and purified according to procedures modified from the literature^32^. In brief, an *E. coli* codon optimized gene coding for the histidine-tagged RfA1 protein from *D. (Loligo) pealeii* (Genbank: ACZ57764.1) was synthesized and cloned into the pJExpress414 vector (ATUM). This expression vector was transformed into BL21 (DE3) cells (Novagen). The protein was expressed in Lysogeny Broth (LB) (Novagen) supplemented with 100 μg/mL Carbenicillin at a temperature of 37 °C. RfA1 was completely insoluble when expressed at 37 °C and was sequestered in inclusion bodies. The cells were lysed using BugBuster (Novagen) according to the manufacturer’s protocols, and the inclusion bodies were extracted by filtration and centrifugation. The inclusion bodies were then solubilized in denaturing buffer (6 M guanidine hydrochloride), and the protein was purified via high performance liquid chromatography (HPLC) on an Agilent 1260 Infinity system using a reverse phase C18 column. For purification, the gradient was evolved from 95% Buffer A:5% Buffer B to 5% Buffer A:95% Buffer B at a flow rate of 4 mL/min over 35 min (Buffer A: 99.9% water, 0.1% trifluoroacetic acid; Buffer B: 95% acetonitrile, 4.9% water, 0.1% trifluoroacetic acid). The pure RfA1 was collected, flash frozen in liquid nitrogen, and lyophilized. The identity of the protein was confirmed with sodium dodecyl sulfate polyacrylamide gel electrophoresis (SDS-PAGE), tryptic digestion, and mass spectrometry, prior to use in any characterization experiments.

### Preparation of human cells for immunofluorescence microscopy

The untransfected or transfected HEK 293 cells were fixed and labeled with fluorescent markers according to standard protocols. First, the cells were seeded on 8-well or 12-well glass-bottom micro-slides (Ibidi) coated with human fibronectin (Corning) at a density of ~30,000 cells/cm^2^ and were grown for ~14 to ~16 h. When necessary, the HEK 293 cells were either (1) transfected with vectors encoding for just RfA1, just RFP, or both RfA1 and RFP for ~48 h (see Growth and transfection of human cells); (2) mock transfected, i.e. subjected to the transfection reagents in the absence of any vector, under the same conditions; or (3) exposed to the FBS-free growth media in the absence of any transfection reagents or vectors under the same conditions. The untransfected or transfected cells were fixed with 3% paraformaldehyde (PFA) in 0.1 M phosphate buffer (PB), permeabilized with 0.1% Triton-X 100 in phosphate buffered saline (PBS) containing 1% bovine serum albumin (BSA), and blocked with 1% BSA in PBS. The fixed untransfected or transfected cells were incubated with either an oligoclonal rabbit anti-histidine-tag primary antibody (ThermoScientific, 710286) solution (prepared at a ratio of 1:500 in PBS containing 1% BSA) or a polyclonal rabbit anti-reflectin primary antibody solution (prepared at a ratio of 1:1000 in PBS containing 1% BSA)^21^. The cells were thoroughly washed with PBS and incubated with a goat anti-rabbit IgG Alexa 488 secondary antibody (ThermoScientific, 11008) solution (prepared at a ratio of 1:250 in PBS containing 1% BSA) and with the nuclear stain 4′,6′-diamidino-2-phenylindole (DAPI) (ThermoScientific). After labeling, the cells were again washed with PBS, and treated with anti-fade mounting media (Ibidi). The resulting stained fixed untransfected and transfected cells were imaged with confocal fluorescence microscopy.

### Preparation of human cells for live/dead assays

The untransfected or transfected HEK 293 cells were labeled with the Calcein AM dye (live cell stain) and the Ethidium Homodimer-1 dye (dead cell stain) according to standard protocols. First, the cells were seeded on 3-well removable chamber glass slides (Ibidi) coated with human fibronectin (Corning) at a density of ~60,000 cells/cm^2^ and were grown for ~14 to ~16 h. When necessary, the HEK 293 cells were transfected with vectors encoding for RfA1 over a period of ~48 h (see Growth and transfection of human cells) or were exposed to the FBS-free growth media in the absence of any transfection reagents or vectors under the same conditions. Next, the untransfected or transfected cells were incubated for ~1 h in MEM supplemented with Earle’s salts, for which the NaCl concentration was adjusted to 117 or 217 mM. In turn, the cells were washed with D-PBS (ThermoScientific) and stained with Calcein AM (ThermoScientific) and Ethidium Homodimer-1 (ThermoScientific) solutions. The resulting stained untransfected and transfected cells were imaged with fluorescence microscopy.

### Preparation of human cells for cell area assays

The untransfected or transfected HEK 293 cells were labeled with fluorescently-tagged wheat germ agglutinin according to standard protocols. First, the cells were seeded on 3-well removable chamber glass slides (Ibidi) coated with human fibronectin (Corning) at a density of ~60,000 cells/cm^2^ and were grown for ~14 to ~16 h. When necessary, the HEK 293 cells were transfected with vectors encoding for RfA1 for ~48 h (see Growth and transfection of human cells) or were exposed to the FBS-free growth media in the absence of any transfection reagents or vectors under the same conditions. Next, the untransfected or transfected cells were incubated for ~1 h in MEM supplemented with Earle’s salts, for which the NaCl concentration was adjusted to 117 mM or 217 mM. In turn, the cells were stained with Alexa 555 fluorophore-conjugated wheat germ agglutinin (ThermoScientific) in Hank’s Balanced Salt Solution (HBSS) (ThermoScientific) and subsequently washed in pre-warmed HBSS. Finally, the cells were fixed with 3% PFA in 0.1 M PB. The resulting stained untransfected and transfected cells were imaged with fluorescence microscopy.

### Brightfield optical microscopy and spectroscopy

The untransfected or transfected HEK 293 cell cultures were integrated into sandwich-type configurations. First, 3-well removable chamber glass slides (Ibidi) were coated with human fibronectin (Corning). Next, HEK 293 cells were seeded at densities of ~60,000 cells/cm^2^ and were grown for ~14 to ~16 h. When necessary, the cells were transfected with vectors encoding for RfA1 for ~48 h (see Growth and transfection of human cells) or were exposed to the FBS-free growth media in the absence of any transfection reagents or vectors under the same conditions. Next, the untransfected or transfected cells were incubated for ~1 h in MEM supplemented with Earle’s salts, for which the NaCl concentration was adjusted to 117 or 217 mM. In turn, the substrates with monolayers at a ~50% to ~75% confluency were fixed with 3% PFA in PBS, thoroughly washed with PBS, treated with anti-fade mounting media (Ibidi), and covered (overlaid) with a thin glass coverslip. Note that the preparation and use of cell cultures within the ~50% to ~75% confluency window ensured rigorous quality control and facilitated comparisons across all of the experiments. The resulting configurations, which contained either fixed transfected or untransfected cells, were imaged with brightfield optical microscopy and characterized with reflectance and transmittance spectroscopy.

### Preparation of cells for transmission electron microscopy

The untransfected or transfected HEK 293 were segmented into cross-sections according to literature protocols^53^. First, HEK 293 cells were seeded at densities of ~32,000 cells/cm^2^ into T-25 flasks (ThermoScientific) and were grown for ~18 to ~24 h. Typically, the HEK 293 cells were transfected with vectors encoding for just RfA1, just RFP, or both RfA1 and RFP for ~48 h in house (see Growth and transfection of human cells) or were exposed to the FBS-free growth media in the absence of any transfection reagents or vectors under the same conditions. Alternatively, for independent confirmation of our experiments, the HEK 293 cells were cultured in Improved MEM supplemented with 10% FBS and were transfected with a reagent mixture containing Fugene HD and the vector encoding for RfA1 at ATUM. Next, the cells were fixed with 2.5% glutaraldehyde in 0.1 M sodium cacodylate buffer (Electron Microscopy Sciences) and spun down into a cell pellet. Subsequently, the pellet was blocked with 1% osmium tetroxide in 0.15 M sodium cacodylate buffer (LADD Research), stained with 2% uranyl acetate in double distilled water (LADD Research), and dehydrated with ethanol (LADD Research). The cells were then embedded in Durcupan resin (Sigma) and sectioned on an Ultracut UC6 Ultramicrotome (Leica) by using a diamond knife (Diatome). The sections were next transferred onto copper mesh grids (LADD Research) and post-stained with uranyl acetate and lead citrate (Electron Microscopy Sciences). The final fixed, resin-embedded, grid-mounted cross-sections were imaged with transmission electron microscopy.

### Preparation of human cells for immuno-electron microscopy

The transfected HEK 293 cells were segmented and labeled with gold nanoparticles according to literature protocols^54,55^. First, HEK 293 cells were seeded at densities of ~32,000 cells/cm^2^ into T-25 flasks (ThermoScientific) and were grown for ~18 to ~24 h. The HEK 293 cells were then transfected with vectors encoding for both RfA1 and RFP (see Growth and transfection of human cells) for ~48 h. Next, the cells were fixed overnight with 4% PFA in 0.1 M PB (Electron Microscopy Sciences), rinsed with 0.15% glycine in 0.1 M PB, pelleted in 10% gelatin in 0.1 M PB, and cryoprotected by infusion with 2.3 M sucrose in 0.1 M PB. Cell blocks with volumes of 1 mm^3^ were then mounted onto cryopins, and flash frozen in liquid nitrogen. The frozen blocks were cut into ~70 to ~90 nm ultra-thin cross-sections at a temperature of –100 °C on an Ultracut UC6 Ultramicrotome with a cryo-attachment (Leica) by using a diamond cryo-knife (Diatome). The sections were in turn picked up with a 1:1 mixture of 2.3 M sucrose in 0.1 M PB and 2% methyl cellulose (Aldrich) in water and transferred onto Formvar and carbon-coated copper grids (Electron Microscopy Sciences). The grid-mounted sections were then placed on 2% gelatin in PBS, rinsed with 0.15% glycine in PBS, and blocked with 1% fish-skin gelatin (Sigma) in PBS. The grid-mounted sections were incubated with a polyclonal rabbit anti-reflectin primary antibody followed by a goat anti-rabbit secondary IgG antibody conjugated to a 12 nm gold nanoparticle (Jackson Immuno Research). The resulting grid-mounted sections were post-fixed with 1% glutaraldehyde in PBS, washed thoroughly with distilled water, and subsequently post-stained with 0.2% uranyl acetate (LADD Research) in 1.8% methyl cellulose in water. The final fixed, resin-embedded, cryoprotected, and labeled cross-sections were imaged with transmission electron microscopy.

### Preparation of human cells for quantitative phase microscopy

The untransfected and transfected HEK 293 cells were grown as described above, with minor modifications to the protocol. In brief, the cells were seeded at a density of ~5000 cells/cm^2^ on glass substrates and grown for ~14 to ~16 h. When necessary, the cells were transfected with vectors encoding for just RfA1 or for both RfA1 and RFP (see Growth and transfection of human cells) immediately prior to imaging. For reflection-mode experiments, the cells were cultured on custom-designed 35 mm glass-bottom dishes featuring an anti-reflection coating^39^, which were coated with human fibronectin (Corning). For transmission-mode experiments, the cells were cultured on custom-designed 35 mm glass-bottom dishes featuring a half-mirror coating, which were coated with human fibronectin^39^. The untransfected or transfected cells were characterized with low-coherence quantitative phase microscopy with or without a fluorescence microscopy attachment.

### Preparation of aqueous reflectin A1 solutions

The solutions were prepared according to procedures adopted from the literature^29,32^. In brief, purified, lyophilized protein was first solubilized in deionized water at a concentration of ~1 to ~4 mg/mL and a low pH of <~5. The protein solution was then diluted to a concentration of ~0.5 mg/mL, and the NaCl concentration was adjusted to 117, 167, 217, or 334 mM as appropriate. The resulting solutions were characterized with transmittance and reflectance spectroscopy and dynamic light scattering.

### Phase contrast and fluorescence microscopy of human cells

The live untransfected or transfected HEK 293 cells were characterized with an Olympus IX51 equipped with an Olympus TH4100 light source, an Olympus U-RFL-T fluorescence laser source, and a QICAM camera. The resulting phase contrast and fluorescence images were analyzed with ImageJ (v 2.0.0-rc-69/1.52i).

### Confocal microscopy of immunolabeled human cells

The fixed and immunolabeled untransfected or transfected HEK 293 cells were characterized with an LSM 780 confocal microscope equipped with a Nikon GaAsP detector and an Argon laser (with fluorophore excitation wavelengths of 405, 458, and 514 nm). The resulting confocal and fluorescence microscopy images were captured with ZEN software and analyzed with ImageJ (v 2.0.0-rc-69/1.52i).

### Fluorescence microscopy of stained human cells

The untransfected or transfected HEK 293 cells stained with the Calcein AM and Ethidium Homodimer-1 dyes were characterized with an EVOS M5000 Imaging System (ThermoScientific) in fluorescence imaging mode. The fixed untransfected or transfected HEK 293 cells labeled with wheat germ agglutinin conjugated to an Alexa Fluor 555 dye were characterized with an EVOS M5000 Imaging System (ThermoScientific) in fluorescence imaging mode. The resulting images were analyzed with ImageJ (v 2.0.0-rc-69/1.52i).

### Transmission electron microscopy of cellular cross-sections

The fixed, resin-embedded, cross-sections from untransfected or transfected HEK 293 cells were characterized with a Tecnai G2 Spirit BioTWIN transmission electron microscope equipped with an Eagle 4k HS digital camera (FEI). The resulting transmission electron microscopy images were analyzed with ImageJ (v 2.0.0-rc-69/1.52i).

### Immuno-electron microscopy of labeled cellular cross-sections

The fixed, resin-embedded, cryoprotected, and labeled cross-sections from transfected HEK 293 cells were characterized with a JEOL 1400Plus transmission electron microscope (JEOL) and outfitted with a OneView 16 megapixel digital camera (Gatan).

### Quantitative phase microscopy of live human cells

The live transfected HEK 293 cells were characterized with a custom-built low-coherence quantitative phase microscope (Hamamatsu). For reflection-mode experiments, the instrument was outfitted with a heating element, a piezo-driven adjustable sample stage (NanoControl), a fluorescence detection module featuring an excitation filter with a center wavelength of 525 nm (Edmund Optics), a high performance long-pass emission filter with a cut-on wavelength of 575 nm (Edmund Optics), and a light-emitting diode with a broadband emission wavelength from 575 to 700 nm. The resulting interference images were analyzed and converted to optical pathlength maps, geometric height maps, and refractive index maps with MATLAB 2017a (MathWorks, Inc.) as previously described^39^. For transmission-mode experiments, the instrument was outfitted with a heating element, an adjustable sample stage (OptoSigma), and a narrowband light-emitting diode with an emission wavelength of 633 nm. The resulting interferences images were analyzed with MATLAB 2017a (MathWorks, Inc.) and converted to phase images and optical pathlength maps as previously described^39^. The phase images and optical pathlength maps were further analyzed with ImageJ to extract the apparent diameter and refractive index of the RfA1-based structures or cytoplasmic regions (note that the accuracy of the size estimates was limited by the use of the 633 nm laser and the resolution of the images and maps).

### Brightfield optical microscopy of human cells

The sandwich-type configurations from untransfected or transfected HEK 293 cells were characterized with an EVOS M5000 Imaging System (ThermoScientific) in brightfield imaging mode. The resulting brightfield optical images were analyzed with ImageJ (v 2.0.0-rc-69/1.52i). The histograms of the number of pixels at different intensity values were extracted from the images and analyzed by using the Histogram function in ImageJ according to standard image processing and analysis procedures reported in the literature^47,48,56^. The histograms were plotted with Igor Pro 6.1.

### Spectroscopy of human cells and reflectin A1 solutions

The sandwich-type configurations containing untransfected or transfected HEK 293 cells, and the RfA1 solutions in quartz cuvettes (Millipore Sigma) were characterized with a V-670 UV-VIS-NIR Spectrophotometer (Jasco) outfitted with a 150 mm Integrating Sphere (Jasco). The obtained spectra were plotted with Igor Pro 6.1

### Dynamic light scattering of reflectin A1 solutions

The RfA1 solutions were characterized with a Zeta-Sizer Nano S (Malvern). The obtained correlograms were analyzed and converted to particle size distributions with the Malvern Panalytical software and plotted with Igor Pro 6.1.

### Statistical analysis

The statistical analyses were performed using Prism v.8 software (GraphPad). Data sets from two samples were compared by applying a Student’s *t*-test to calculate two-tailed *p*-values.

### Overview of statistics and reproducibility

Representative immunofluorescence images from *n* = 5 biological replicates are shown in Fig. 2a. Representative transmission electron microscopy images from *n* = 4 biological replicates are shown in Fig. 2b. Representative immunofluorescence images from *n* = 5 biological replicates are shown in Fig. 2c. Representative immuno-electron microscopy images from *n* = 3 technical replicates are shown in Fig. 2d. Representative phase images from *n* = 6 biological replicates are shown in Fig. 3a. Representative phase images, fluorescence images, and refractive index maps from *n* = 5 biological replicates are shown in Fig. 3b. Representative phase images and optical pathlength maps from *n* = 4 biological replicates are shown in Fig. 3c. Representative brightfield microscopy images from *n* = 5 biological replicates are shown in Fig. 4a. Representative diffuse transmittance spectra and diffuse reflectance spectra from at least *n* = 5 biological replicates are shown Fig. 4b. Representative digital camera images from *n* = 6 biological replicates are shown in Fig. 4c. Representative diffuse transmittance spectra and diffuse reflectance spectra from at least *n* = 5 biological replicates are shown Fig. 4d.

### Reporting summary

Further information on research design is available in the Nature Research Reporting Summary linked to this article.

## Supplementary information

Supplementary Information

Supplementary Movie 1

Supplementary Movie 2

Description of Additional Supplementary Files

Reporting Summary

## Data Availability

All data needed to evaluate the conclusions in the paper are present in the paper and/or the supplementary information. Source data are available in the Source Data file. All other relevant data are available from the authors upon reasonable request.

## Source data

Source Data
