# Supplementary material for: Cephalopod-inspired optical engineering of human cells
Source: Nat Commun. 2020 Jun 2;11:2708. doi: 10.1038/s41467-020-16151-6 (PMC7266819; doi:10.1038/s41467-020-16151-6)
Supplement: Supplementary file 1 — Supplementary Information [file 41467_2020_16151_MOESM1_ESM.pdf]

Supplementary Information for

Cephalopod-Inspired Optical Engineering of Human Cells

Chatterjee *et al.*

Supplementary Figures:

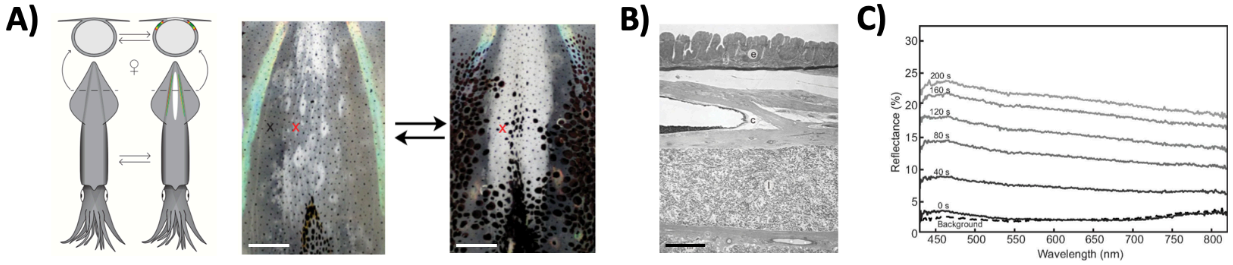

Supplementary Figure 1: The functionality and architecture of a female *D. opalescens* squid's leucophore-containing tissues. (**A**) (Left) An illustration of a female *Doryteuthis opalescens* squid that switches a white stripe on its mantle from nearly transparent (left) to opaque white (right). (Right) Photographs of the white stripe on the mantle of a female *D. opalescens* squid. The stripe switches from nearly transparent (left) to opaque white (right) after the injection of acetylcholine. The scale bar is 0.5 cm. (**B**) A light micrograph of a semi-thin section of the female white stripe on a *D. opalescens* squid that shows the tissue strata, i.e. the epidermis (e), chromatophore layer (c), thick female-specific leucophore layer (l) and underlying muscle (m). The scale bar is 100 µm. (**C**) A plot of the reflectance as a function of wavelength obtained for the white stripe on the mantle of a female *D. opalescens* squid at different time points after the injection of acetylcholine. The broadband reflectance progressively increases with time. [The cartoon, photographs, and plot were reproduced from (*1*) with the permission of the publisher.]

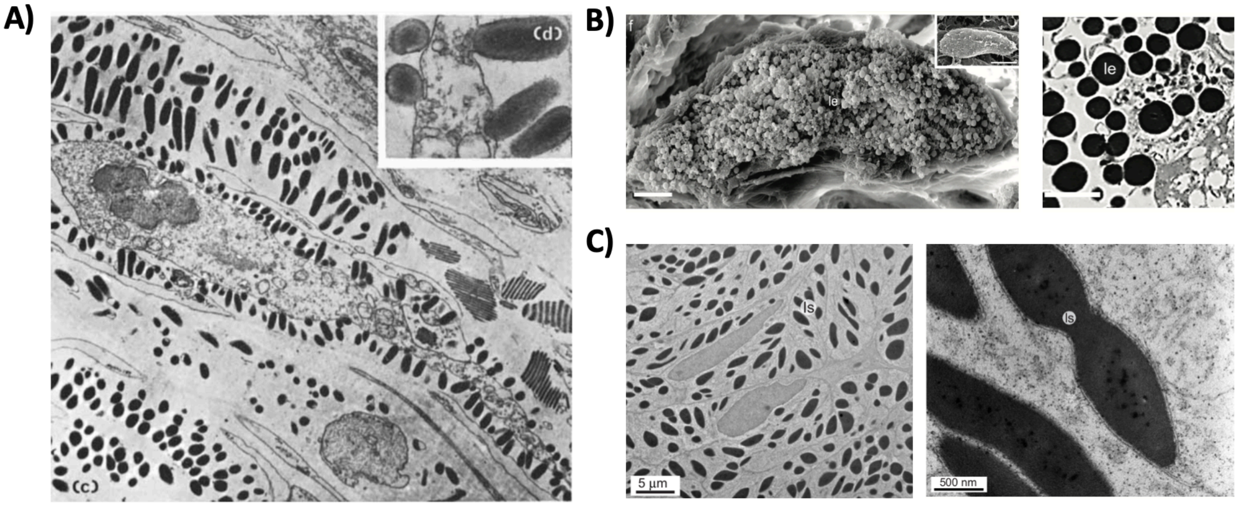

Supplementary Figure 2: Electron microscopy images of cephalopod leucophores. (**A**) A TEM image of a cross-section from an *Octopus vulgaris* leucophore, which shows the electron-dense, membrane bound leucosomes at the cellular periphery (also see the inset for a magnified view). The magnification is 5000 X (25,000 X for the inset). There is no associated scale bar for this image. [The images were reproduced from (*2*) with the permission of the publisher.] (**B**) (Left) An SEM image of a fractured cuttlefish leucophore, wherein the cell body contains a disordered arrangement of leucosomes (also see the inset for an intact cell). The scale bar is 2 μm. (Right) A TEM image of a cross-section from a *Sepia officinalis* leucophore, which shows electron-dense leucosomes associated with cytoplasmic endoplasmic reticulum strands. The scale bar is 2 μm. [The images were reproduced from (*3*) with the permission of the publisher.] (**C**) (Left) A TEM image of the leucophore layer from the white stripe of a female *Doryteuthis opalescens* squid. (Right) A TEM image of leucosomes (dark gray) within the leucophores. [The images were reproduced from (*1*) with the permission of the publisher.]

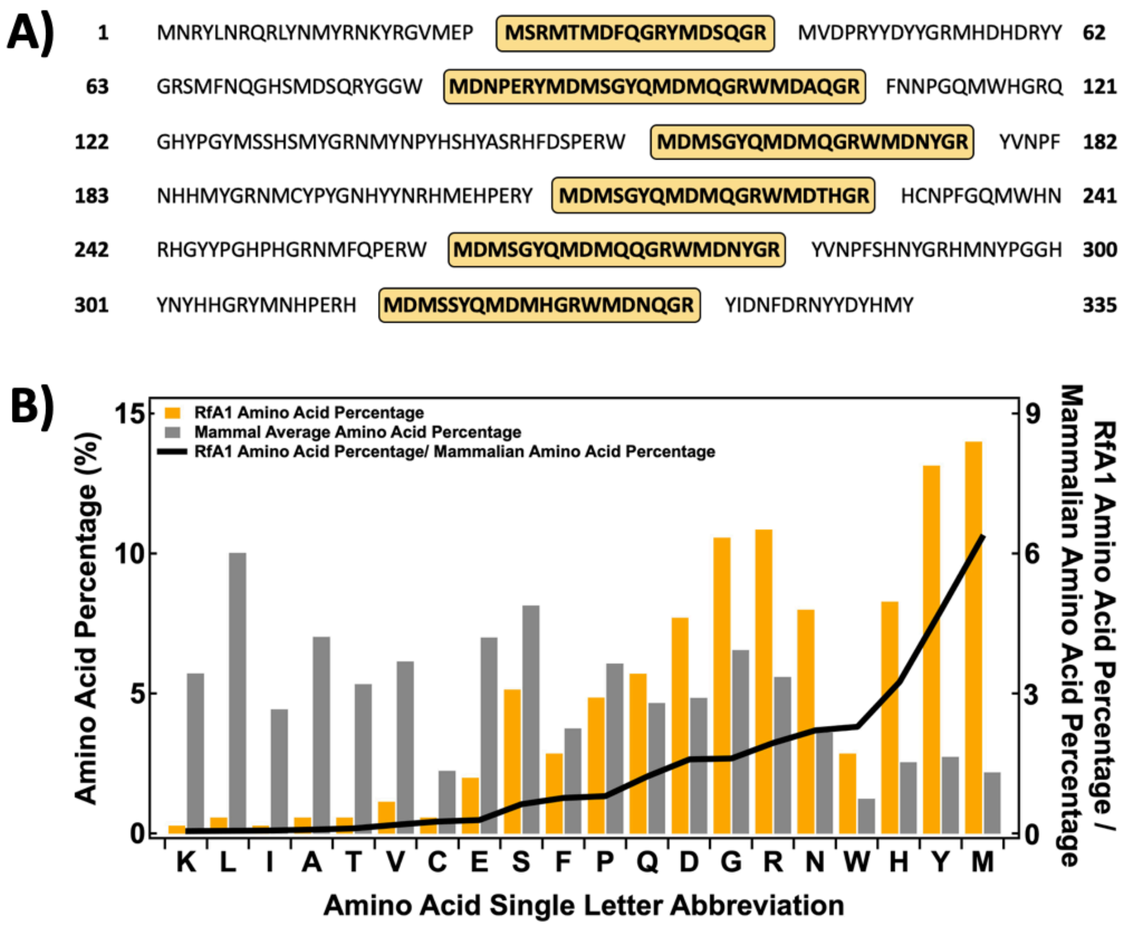

Supplementary Figure 3: The wild type *Doryteuthis pealeii* reflectin A1 sequence and a comparison of the percentage of each amino acid in this sequence with the percentage of each amino acid in mammalian proteins. (**A**) The amino acid sequence for the *D. pealeii* RfA1 isoform, with the general conserved motifs indicated by the orange boxes. (**B**) A comparison of the percentage of each amino acid in the RfA1 protein sequence (orange bars) and the average percentage of each amino acid in mammalian proteins (gray bars), along with the ratio of these two percentages for each amino acid (black line). The average percentages of each amino acid for mammalian proteins were obtained from the UniProt database (*4*).

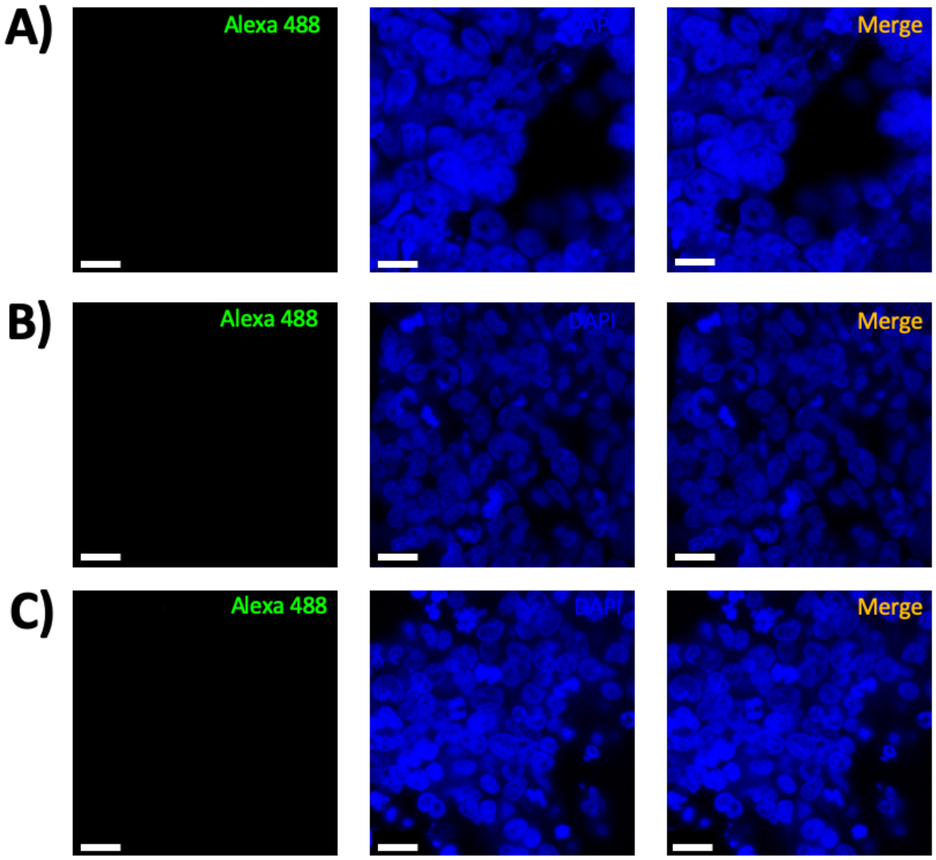

Supplementary Figure 4: Fluorescence microscopy images of human cells transfected with a vector encoding for the expression of reflectin A1. (**A**) Fluorescence microscopy images of fixed RfA1-expressing cells stained with DAPI and labeled with only the Alexa 488 fluorophore-conjugated secondary antibody. (Left) There are no signals corresponding to the Alexa 488 fluorophore-conjugated secondary antibody on the relevant channel. (Middle) The signals corresponding to DAPI on the relevant channel are colored blue. (Right) The merged signals observed for the Alexa 488 fluorophore-conjugated secondary antibody and DAPI are shown. The scale bars are 20 µm. (**B**) Fluorescence microscopy images of fixed RfA1-expressing cells stained with DAPI and labeled with only a histidine tag-specific primary antibody. (Left) There are no signals corresponding to the Alexa 488 fluorophore-conjugated secondary antibody on the relevant channel. (Middle) The signals corresponding to DAPI on the relevant channel are colored blue. (Right) The merged signals observed for the Alexa 488 fluorophore-conjugated secondary antibody and DAPI are shown. The scale bars are 20 µm. (**C**) Fluorescence microscopy images of fixed RfA1-expressing cells stained with DAPI and labeled with only the reflectin sequence-specific primary antibody. (Left) There are no signals corresponding to the Alexa 488 fluorophore-conjugated secondary antibody on the relevant channel. (Middle) The signals corresponding to DAPI on the relevant channel are colored blue. (Right) The merged signals observed for the Alexa 488 fluorophore-conjugated secondary antibody and DAPI are shown. The scale bars are 20 µm. Representative immunofluorescence microscopy images from n = 5 biological replicates are shown.

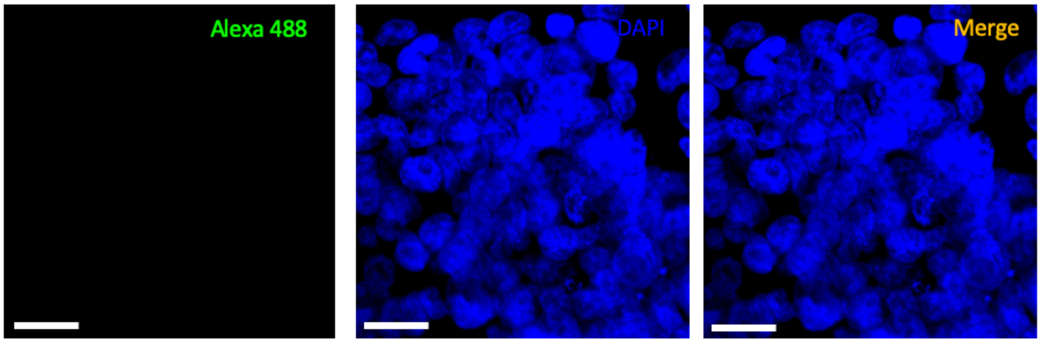

Supplementary Figure 5: Fluorescence microscopy images of mock transfected human cells. Fluorescence microscopy images of fixed mock transfected cells, i.e. ones treated with only with the transfection reagents in the absence of any vector, which were stained with DAPI and labeled with an antibody pair specific for reflectins' unique sequence. (Left) There are no signals corresponding to the Alexa 488 fluorophore-conjugated secondary antibody on the relevant channel. (Middle) The signals corresponding to DAPI on the relevant channel are colored blue. (Right) The merged signals observed for the Alexa 488 fluorophore-conjugated secondary antibody and DAPI are shown. The scale bars are 20 µm. Representative immunofluorescence microscopy images from $n = 3$ biological replicates are shown.

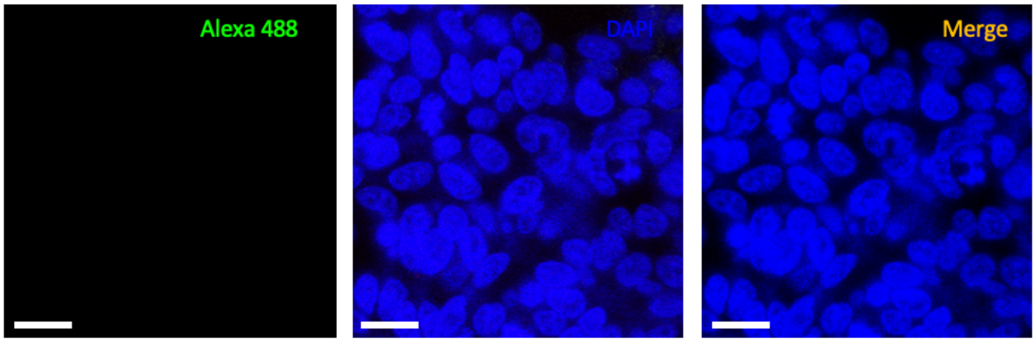

Supplementary Figure 6: Fluorescence microscopy images of untransfected human cells. Fluorescence microscopy images of fixed untransfected cells stained with DAPI and labeled with an antibody pair specific for reflectins' unique sequence. (Left) There are no fluorescence signals corresponding to the Alexa 488 fluorophore-conjugated secondary antibody on the relevant channel. (Middle) The fluorescence signals corresponding to DAPI on the relevant channel are colored blue. (Right) The merged fluorescence signals observed for the Alexa 488 fluorophore-conjugated secondary antibody and DAPI are shown. The scale bars are 20 μm. Representative immunofluorescence microscopy images from n = 5 biological replicates are shown.

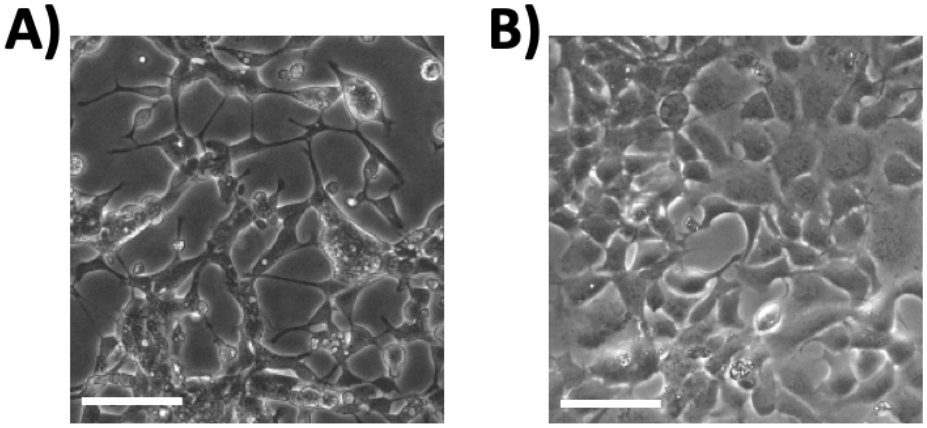

Supplementary Figure 7: Phase contrast images of transfected and untransfected human cells. (**A**) A phase contrast image of live RfA1-expressing cells. The scale bar is 25 µm. (**B**) A phase contrast image of untransfected cells. The scale bar is 25 µm. Representative phase contrast microscopy images from *n* = 6 biological replicates are shown.

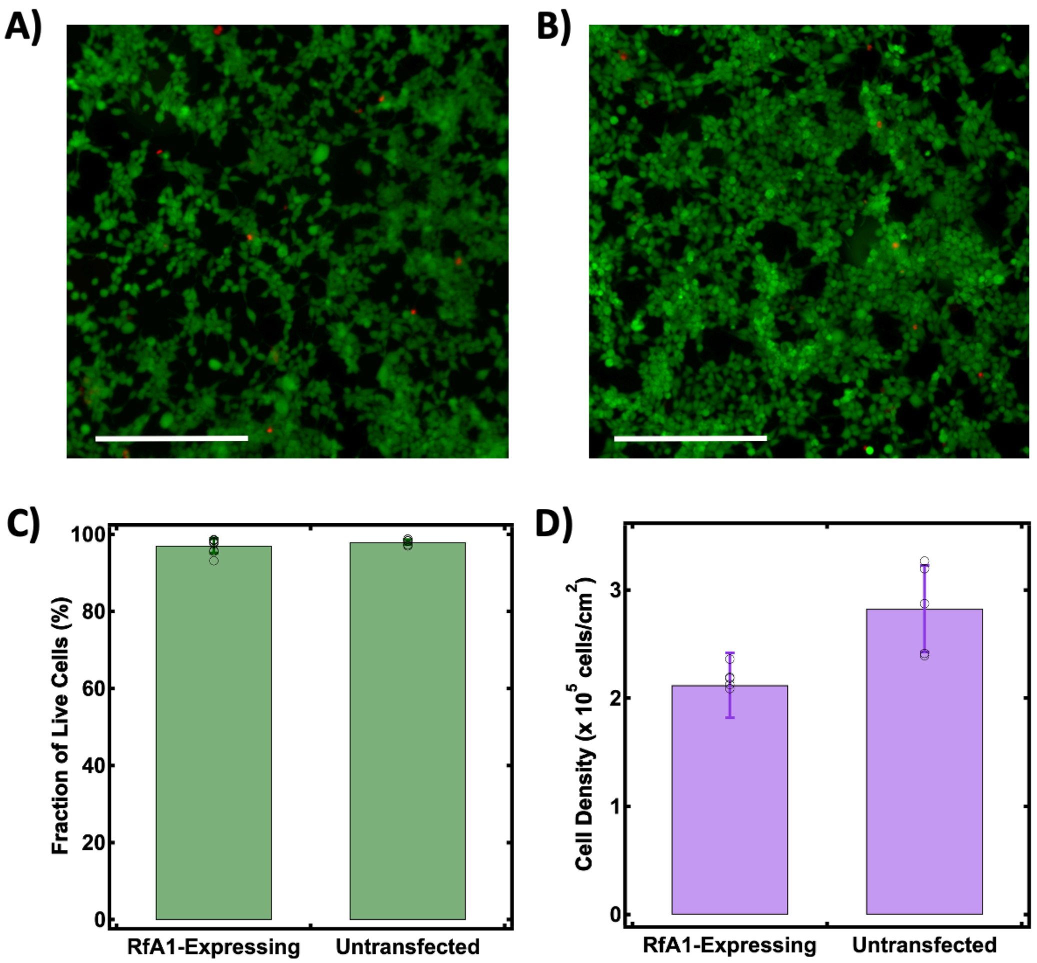

Supplementary Figure 8: Fluorescence microscopy images and plots for the viabilities and cell densities of transfected and untransfected human cells. (**A**) Merged fluorescence microscopy images of live RfA1-expressing cells stained with the live cell-specific calcein AM fluorescent dye and the dead cell-specific ethidium homodimer-1 fluorescent dye. The signals corresponding to the calcein AM and ethidium homodimer-1 dyes are colored green and red, respectively. The scale bar is 400 µm. (**B**) The merged fluorescence microscopy images of live untransfected cells stained with the live cell-specific calcein AM fluorescent dye and the dead cell-specific ethidium homodimer-1 fluorescent dye. The signals corresponding to the calcein AM and ethidium homodimer-1 dyes are colored green and red, respectively. The scale bar is 400 µm. (**C**) A plot of the fraction of live cells for the RfA1-expressing and untransfected cells. (**D**) A plot of the cell density for RfA1-expressing and untransfected cells. Representative fluorescence microscopy images from $n = 3$ biological replicates are shown in (**A**) and (**B**). The fractions of live cells in (**C**) and the cell densities in (**D**) were calculated by analyzing $n = 5$ randomly selected fields of view from the $n = 3$ biological replicates. The bar plots correspond to the mean values and the error bars correspond to the standard deviations.

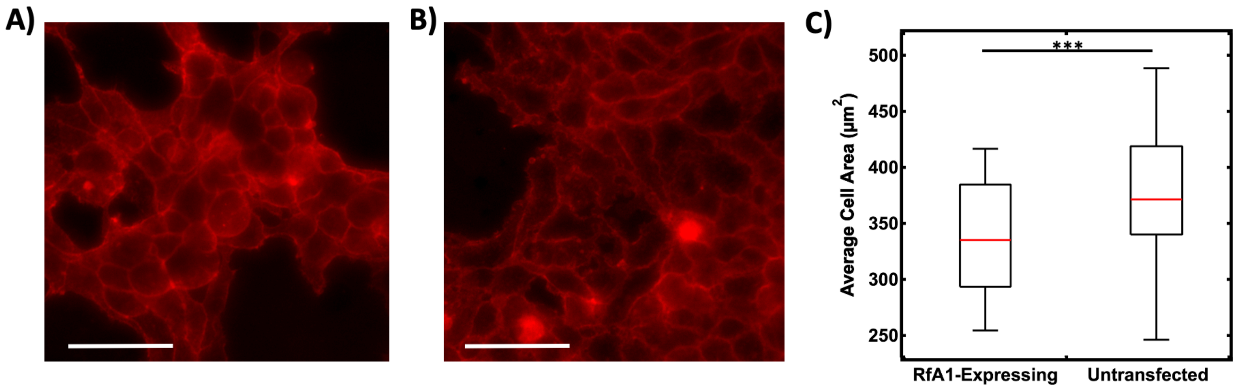

Supplementary Figure 9: Fluorescence microscopy images and plots for the average areas of transfected and untransfected human cells. (**A**) A fluorescence microscopy image of fixed RfA1-expressing cells stained with Alexa 555 fluorophore-conjugated wheat germ agglutinin. The corresponding fluorescence signals are colored red. The scale bar is 50 µm. (**B**) A fluorescence microscopy image of fixed untransfected cells stained with Alexa 555 fluorophore-conjugated wheat germ agglutinin. The corresponding fluorescence signals are colored red. The scale bar is 50 µm. (**C**) Box-and-whisker plots of the average cell areas for RfA1-expressing and untransfected cells. The box represent the first and third quartiles, the whiskers represent the minimum and maximum values, and the red lines represent the mean values. Note that the calculated areas were typical of > 90 % of the transfected and untransfected cells within each field of view, with the remaining cells featuring areas that were larger and/or challenging to estimate accurately. Representative fluorescence microscopy images from n = 3 biological replicates are shown in (**A**) and (**B**). The cell areas that were calculated for n = 60 cells from the n = 3 biological replicates are shown in (**C**). A two-sided Student's t test was performed, wherein $P < 0.05$ was considered to be statistically significant and *** corresponds to $P = 0.0001$.

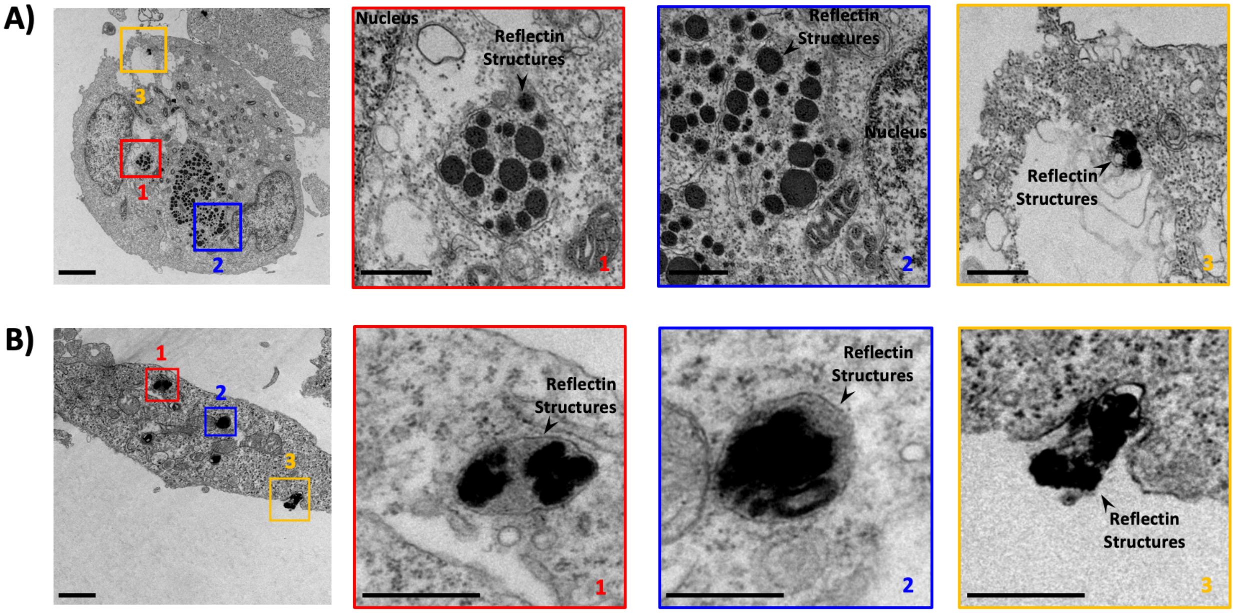

Supplementary Figure 10: Transmission electron microscopy images of human cells transfected with a vector encoding for the expression of reflectin A1. (**A**) (Left) A TEM image of a cross-section from one RfA1-expressing cell, which reveals the presence of electron-dense structures. The scale bar is 2 μm. (Right) The corresponding insets show close-up images of a spheroidal nanoparticle cluster next to cytoplasmic vesicles and the nucleus (red), a spheroidal nanoparticle cluster next to a mitochondrion and the nucleus (blue), and an irregular nanostructure next to a large cytoplasmic vesicle (orange). The scale bars are 500 nm for the insets. (**B**) (Left) A TEM image of a cross-section from another RfA1-expressing cell, which reveals the presence of irregular nanostructures. The scale bar is 1 μm. (Right) The corresponding insets show close-up images of two irregular nanostructures next to the cell's periphery/membrane (red), an irregular nanostructure next to a large cytoplasmic vesicle (blue), and an irregular nanostructure presumably captured in the process of being expelled from the cell (orange). The scale bars are 500 nm for the insets. Representative TEM images from $n = 4$ biological replicates are shown.

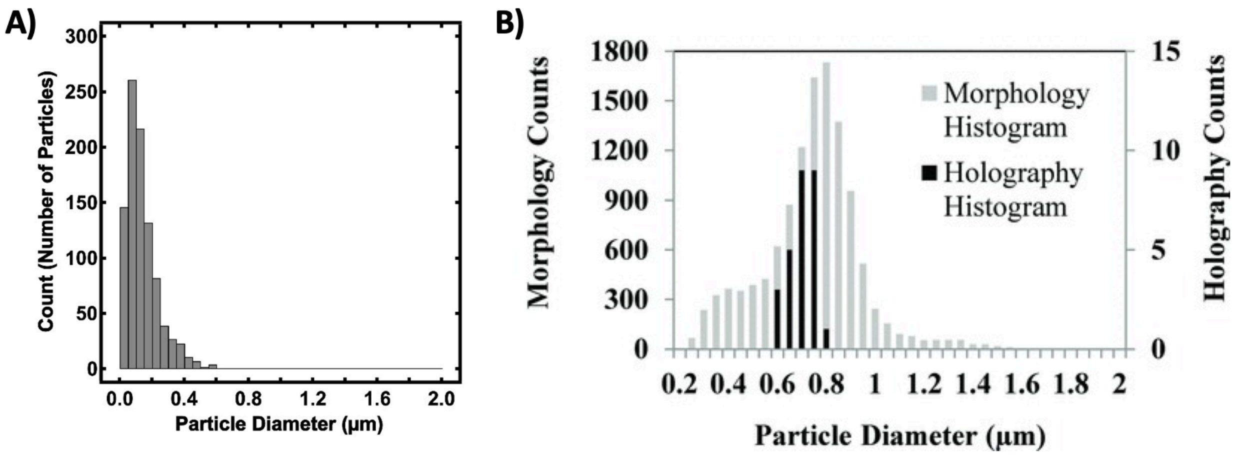

Supplementary Figure 11: Size distributions for the particles found in human cells transfected with a vector encoding for the expression of reflectin A1 and for the particles found in cuttlefish leucosomes. (**A**) A plot of the size distribution for electron-dense particles (structures) in human cells transfected with a vector encoding for the expression of RfA1. The particle (structure) sizes were calculated from *n* = 25 representative TEM images, which were obtained from *n* = 4 biological replicates. The sizes were binned to the nearest 0.05 µm. (**B**) A plot of the particle size distribution for *Sepia officinalis* leucophores. The plot was reproduced from (*5*) with the permission of the publisher.

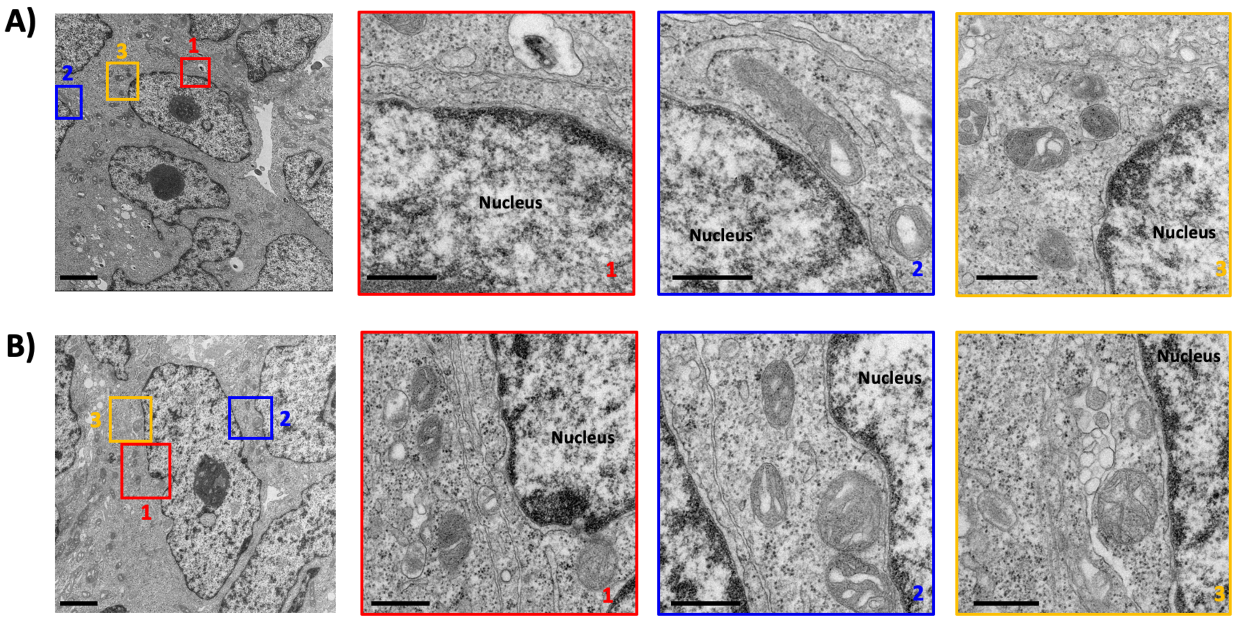

Supplementary Figure 12: Transmission electron microscopy images of untransfected human cells. (**A**) (Left) A TEM image of a cross-section from untransfected cells, which only reveals the presence of various organelles, e. g. nuclei, mitochondria, and ribosomes. The scale bar is 2 µm. (Right) The corresponding insets show close-up images of a likely vesicle adjacent to a nucleus (red), various organelles adjacent to a nucleus (blue), and likely mitochondria adjacent to a nucleus (orange). The scale bars are 500 nm for the insets. (**B**) (Left) A TEM image of a cross-section from a different untransfected cell, which only reveals the presence of various organelles, e. g. nuclei, mitochondria, and ribosomes. The scale bar is 1 µm. (Right) The corresponding insets show close-up images of vesicles and organelles adjacent to a nucleus (red), likely mitochondria between two nuclei (blue), and various organelles adjacent to a nucleus (orange). The scale bars are 500 nm for the insets. Representative TEM images from $n = 4$ biological replicates are shown.

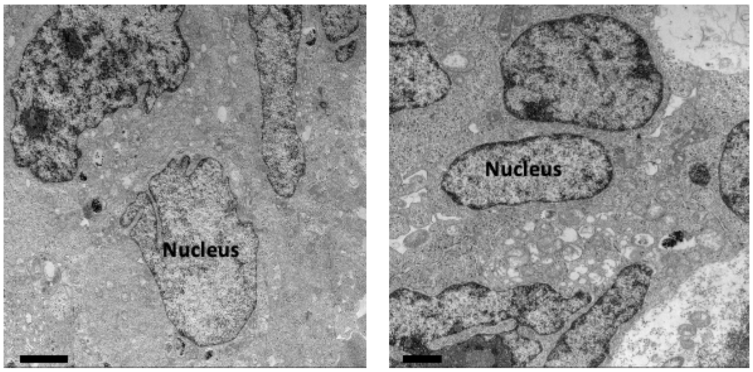

Supplementary Figure 13: Transmission electron microscopy images of human cells transfected with a vector encoding for the expression of red fluorescent protein. Two TEM images of different cross-sections from RFP-expressing cells, which reveals the presence of various organelles. The scale bars are 2 µm (left) and 1 µm (right). Representative TEM images from n = 2 biological replicates are shown.

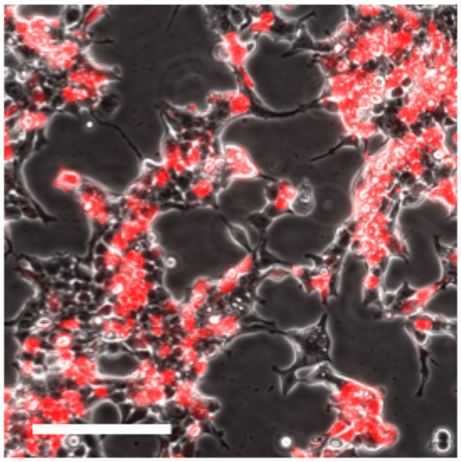

Supplementary Figure 14: Overlaid phase contrast and fluorescence microscopy images of human cells transfected with a vector encoding for the expression of both reflectin A1 and red fluorescent protein. Overlaid phase contrast and fluorescence microscopy images obtained for live RfA1- and RFP-expressing cells. The fluorescence signals corresponding to RFP are colored red. The scale bar is 100 µm. Representative phase contrast and fluorescence microscopy images from $n = 3$ biological replicates are shown.

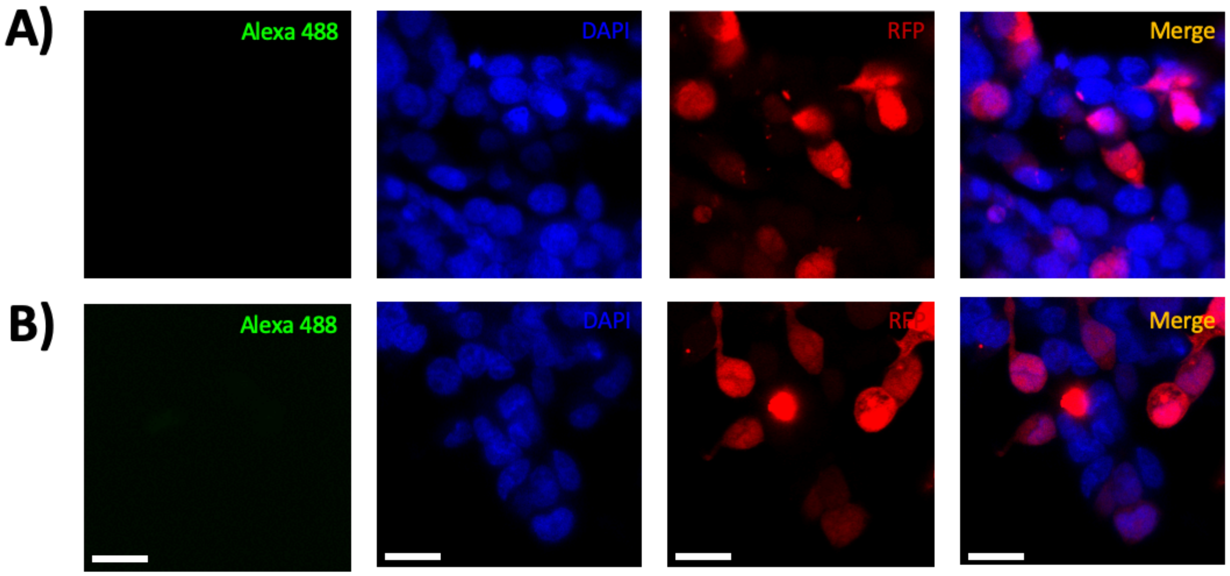

Supplementary Figure 15: Fluorescence microscopy images of human cells transfected with a vector encoding for the expression of both reflectin A1 and red fluorescent protein. (**A**) Fluorescence microscopy images of fixed RfA1- and RFP-expressing cells stained with DAPI and labeled with only the Alexa 488 fluorophore-conjugated secondary antibody. (Left) There are no signals corresponding to the Alexa 488 fluorophore-conjugated secondary antibody on the relevant channel. (Middle Left) The signals corresponding to DAPI on the relevant channel are colored blue. (Middle Right) The signals corresponding to RFP on the relevant channel are colored red. (Right) The merged signals observed for the Alexa 488 fluorophore-conjugated secondary antibody, DAPI, and RFP are shown. The scale bars are 20 μm. (**B**) Fluorescence microscopy images of fixed RfA1- and RFP-expressing cells stained with DAPI and labeled with only the reflectin sequence-specific primary antibody. (Left) There are no signals corresponding to the Alexa 488 fluorophore-conjugated secondary antibody on the relevant channel. (Middle Left) The signals corresponding to DAPI on the relevant channel are colored blue. (Middle Right) The signals corresponding to RFP on the relevant channel are colored red. (Right) The merged signals observed for the Alexa 488 fluorophore-conjugated secondary antibody, DAPI, and RFP are shown. The scale bars are 20 μm. Representative immunofluorescence images from n = 5 biological replicates are shown.

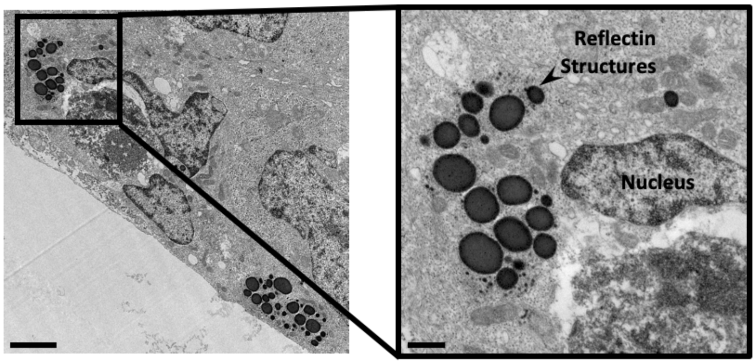

Supplementary Figure 16: Transmission electron microscopy of human cells transfected with a vector encoding for the expression of both reflectin A1 and red fluorescent protein. (Left) A TEM image of a cross-section from RfA1- and RFP-expressing cells, which reveals the presence of electron-dense spheroidal nanoparticles. (Right) The corresponding inset shows a close-up image of a cluster of spheroidal nanoparticles next to cytoplasmic vesicles and the nucleus. The scale bars are 2 μm (left) and 500 nm (right). Representative TEM images from n = 3 biological replicates are shown.

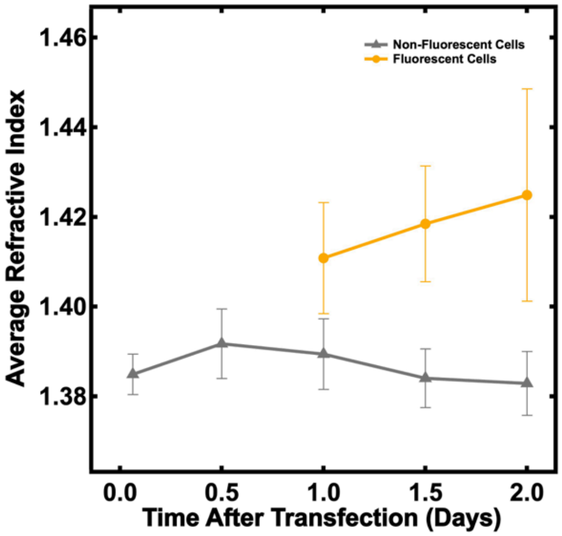

Supplementary Figure 17: Average refractive indices for human cells that either express both reflectin A1 and red fluorescent protein or do not express either biomolecule. A plot of the average refractive index as a function of time for cells after transfection with a vector encoding for both RfA1 and RFP. The average refractive indices of non-fluorescent cells that did not express both RfA1 and RFP are indicated in gray, and the average refractive indices of fluorescent cells that did express both proteins are indicated in orange. The average refractive indices were calculated by analyzing n = 5 randomly selected fields of view from n = 3 biological replicates. The data points correspond to the mean values and the error bars correspond to the standard deviations.

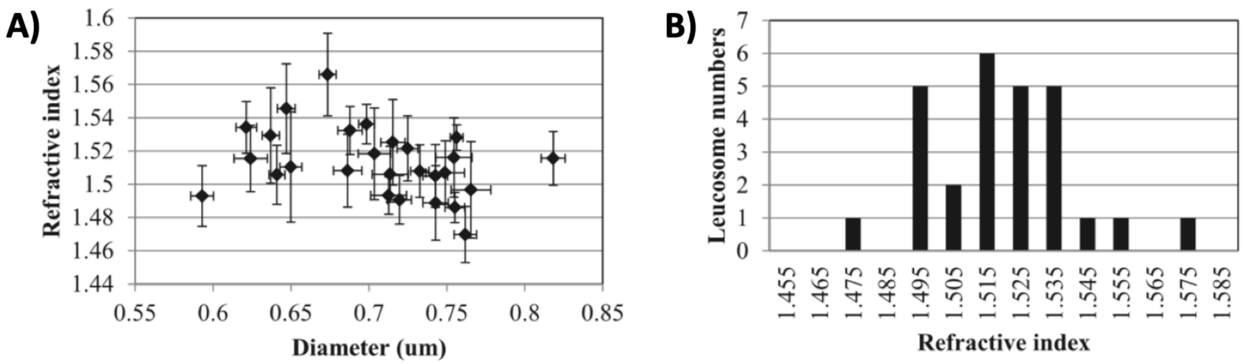

Supplementary Figure 18: Refractive index distributions for leucosomes from *Sepia officinalis* leucophores. (**A**) A plot of the measured refractive index as a function of the leucosome diameter for *Sepia officinalis* cuttlefish leucophores. (**B**) A histogram of the number of leucosomes as a function of the refractive index for *Sepia officinalis* cuttlefish leucophores. The plots were reproduced from (*5*) with the permission of the publisher.

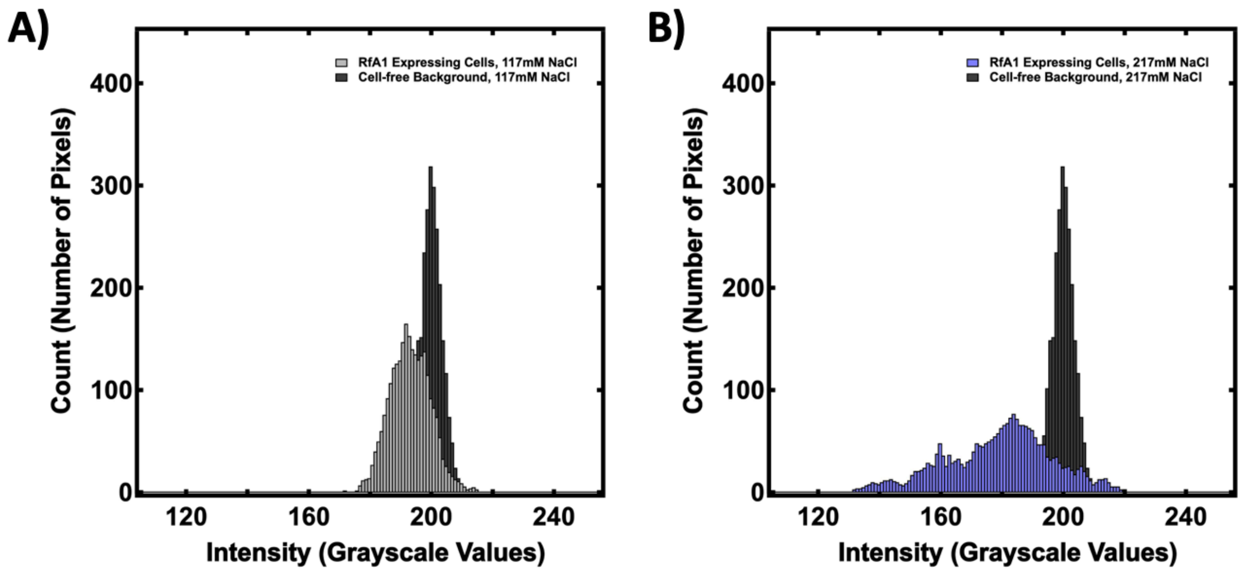

Supplementary Figure 19: Histograms of the pixel count versus intensity for human cells transfected with a vector encoding for the expression of reflectin A1. (**A**) A representative histogram of the count (number of pixels) as a function of the intensity (grayscale values) for RfA1-expressing cells in a sandwich-type configuration after exposure to media with a standard, i.e. 117 mM, NaCl concentration (gray bars). An analogous representative histogram obtained for the cell-free background in the same sandwich-type configuration after exposure to media with a standard, i.e. 117 mM, NaCl concentration (black bars). (**B**) A representative histogram of the count (number of pixels) as a function of the intensity (grayscale values) for RfA1-expressing cells in a sandwich-type configuration after exposure to media with a higher, i.e. 217 mM, NaCl concentration (gray bars). An analogous representative histogram obtained for the cell-free background in the same sandwich-type configuration after exposure to media with a higher, i.e. 217 mM, NaCl concentration (black bars).

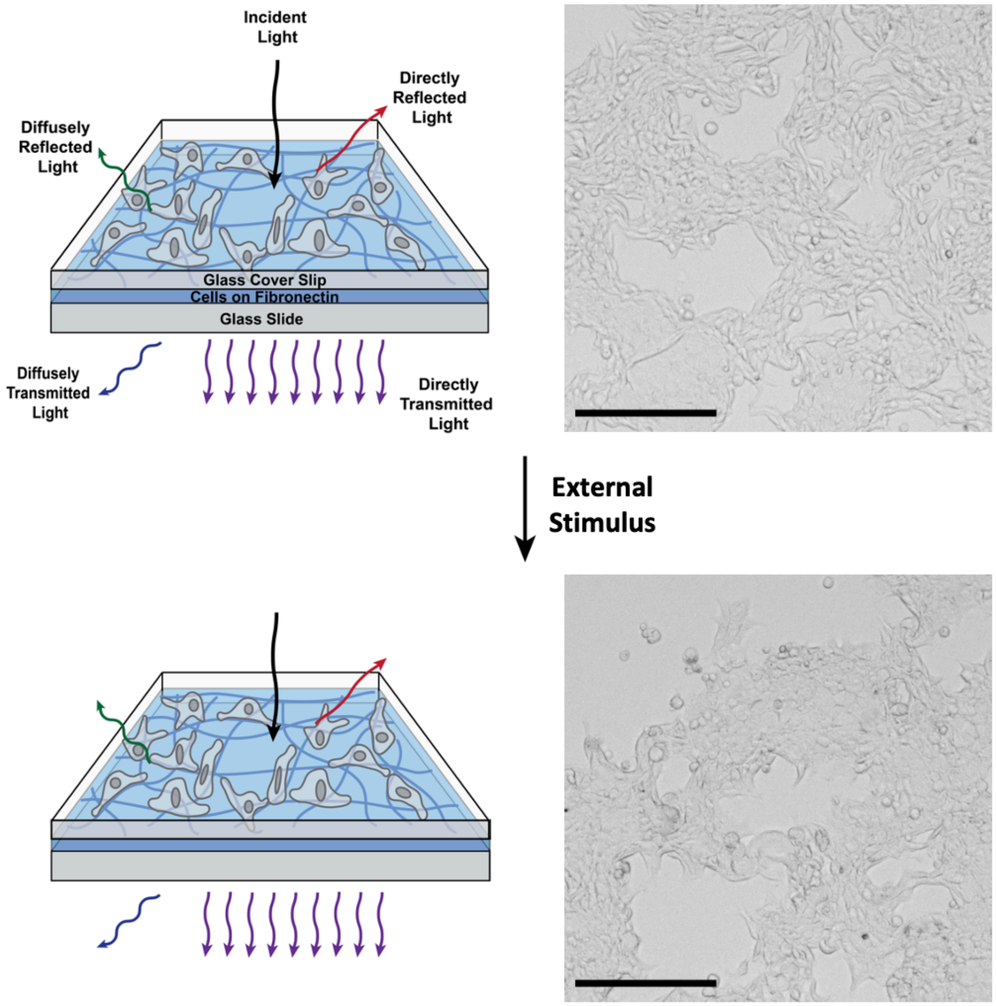

Supplementary Figure 20: Brightfield microscopy images of untransfected human cells. (Top) A schematic (left) and representative brightfield microscopy image (right) of a sandwich-type configuration, wherein the middle layer consists of an untransfected cell culture, after exposure to media with a standard, i.e. 117 mM, NaCl concentration. The cells do not substantially attenuate incident light and appear similar to their environment. (Bottom) A schematic (left) and representative brightfield microscopy image (right) of a sandwich-type configuration, wherein the middle layer consists of an untransfected cell culture, after exposure to media with a higher, i.e. 217 mM, NaCl concentration. The cells do not strongly attenuate light and again appear similar to their environment. The scale bars are 200 μm. Representative brightfield images from n = 5 biological replicates are shown.

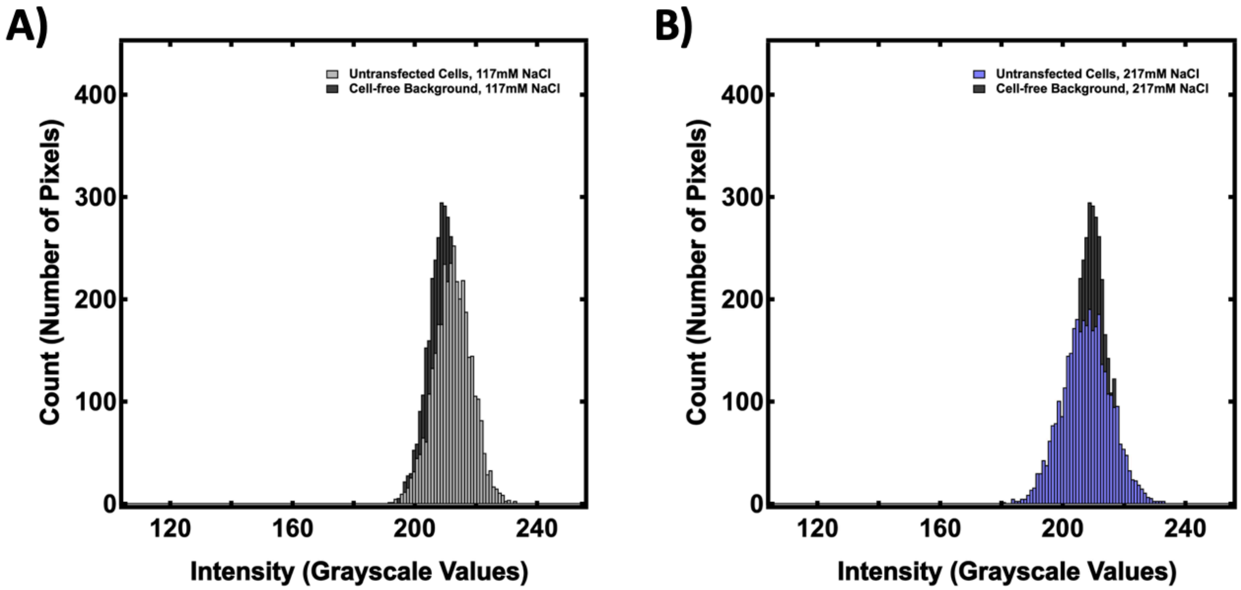

Supplementary Figure 21: Histograms of the pixel count versus intensity for untransfected human cells. (**A**) A representative histogram of the count (number of pixels) as a function of the intensity (grayscale values) for untransfected cells in a sandwich-type configuration after exposure to media with a standard, i.e. 117 mM, NaCl concentration (gray bars). An analogous representative histogram obtained for the cell-free background in the same sandwich-type configuration after exposure to media with a standard, i.e. 117 mM, NaCl concentration (black bars). (**B**) A representative histogram of the count (number of pixels) as a function of the intensity (grayscale values) for untransfected cells in a sandwich-type configuration after exposure to media with a higher, i.e. 217 mM, NaCl concentration (gray bars). An analogous representative histogram obtained for the cell-free background in the same sandwich-type configuration after exposure to media with a higher, i.e. 217 mM, NaCl concentration (black bars).

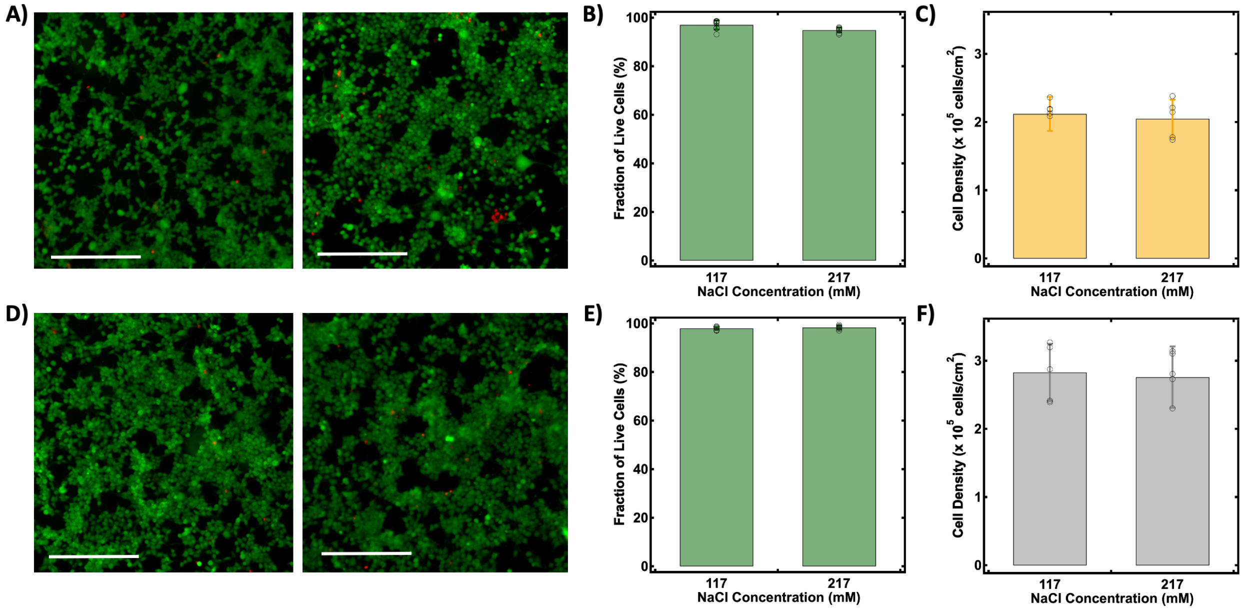

Supplementary Figure 22: Fluorescence microscopy images and plots for the viabilities and cell densities of transfected and untransfected human cells. (**A**) (Left) Merged fluorescence microscopy images of live RfA1-expressing cells, which were stained with the live cell-specific calcein AM fluorescent dye and the dead cell-specific ethidium homodimer-1 fluorescent dye, after exposure to media with a standard, i.e. 117 mM, NaCl concentration. The signals corresponding to the calcein AM and ethidium homodimer-1 dyes are colored green and red, respectively. (Right) Merged fluorescence microscopy images of live RfA1-expressing cells, which were stained with the live cell-specific calcein AM fluorescent dye and the dead cell-specific ethidium homodimer-1 fluorescent dye, after exposure to media with a higher, i.e. 217 mM, NaCl concentration. The signals corresponding to the calcein AM and ethidium homodimer-1 dyes are colored green and red, respectively. The scale bars are 400 µm. (**B**) A plot of the fraction of live cells as a function of the NaCl concentration for the RfA1-expressing cells. (**C**) A plot of the cell density as a function of the NaCl concentration for the RfA1-expressing cells. (**D**) (Left) Merged fluorescence microscopy images of live untransfected cells, which were stained with the live cell-specific calcein AM fluorescent dye and the dead cell-specific ethidium homodimer-1 fluorescent dye, after exposure to media with a standard, i.e. 117 mM, NaCl concentration. The signals corresponding to the calcein AM and ethidium homodimer-1 dyes are colored green and red, respectively. (Right) Merged fluorescence microscopy images of live untransfected cells, which were stained with the live cell-specific calcein AM fluorescent dye and the dead cell-specific ethidium homodimer-1 fluorescent dye, after exposure to media with a higher, i.e. 217 mM, NaCl concentration. The signals corresponding to the calcein AM and ethidium homodimer-1 dyes are colored green and red, respectively. The scale bars are 400 µm. (**E**) A plot of the fraction of live cells as a function of the NaCl concentration for the untransfected cells. (**F**) A plot of the cell density as a function of the NaCl concentration for the untransfected cells. Representative fluorescence images from $n = 3$ biological replicates are shown in (**A**) and (**D**). The fractions of live cells in (**B**) and (**E**) and the cell densities in (**C**) and (**F**) were calculated by analyzing $n = 5$ randomly selected fields of view from the $n = 3$ biological replicates. The bar plots correspond to the mean values and the error bars correspond to the standard deviations. [The same representative images and plots corresponding to transfected cells and untransfected cells exposed to the standard NaCl concentration are also shown in Figure S8.]

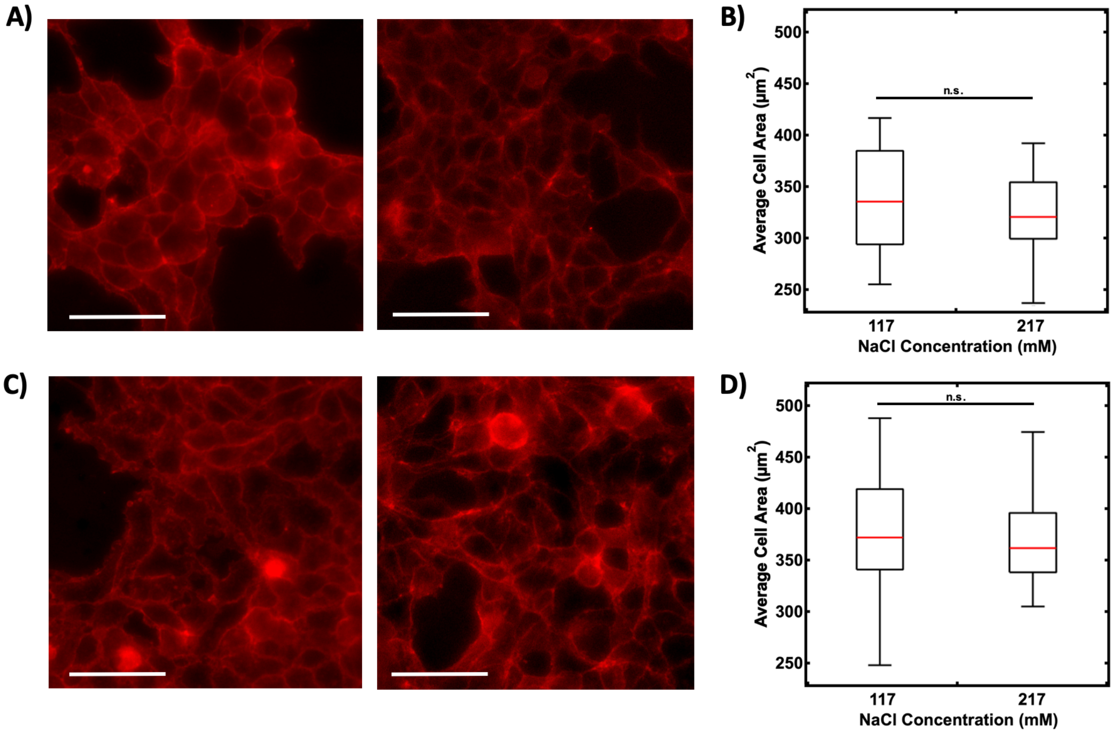

Supplementary Figure 23: Fluorescence microscopy images and plots for the average areas of the transfected and untransfected human cells. (**A**) (Left) A fluorescence microscopy image of fixed RfA1-expressing cells stained with Alexa 555 fluorophore-conjugated wheat germ agglutinin after exposure to media with a standard, i.e. 117 mM, NaCl concentration. The corresponding signals are colored red. (Right) A fluorescence microscopy image of fixed RfA1-expressing cells stained with Alexa 555 fluorophore-conjugated wheat germ agglutinin after exposure to media with a higher, i.e. 217 mM, NaCl concentration. The corresponding signals are colored red. The scale bars are 50 µm. (**B**) Box-and-whisker plots of the average cell areas as a function of the NaCl concentration for RfA1-expressing cells. The boxes represent the first and third quartiles, the whiskers represent the minimum and maximum values, and the red lines represent the mean value. (**C**) (Left) A fluorescence microscopy image of fixed untransfected cells stained with Alexa 555 fluorophore-conjugated wheat germ agglutinin after exposure to media with a standard, i.e. 117 mM, NaCl concentration. The corresponding signals are colored red. (Right) A fluorescence microscopy image of fixed untransfected cells stained with Alexa 555 fluorophore-conjugated wheat germ agglutinin after exposure to media with a higher, i.e. 217 mM, NaCl concentration. The corresponding signals are colored red. The scale bars are 50 µm. (**D**) Box-and-whisker plots of the average cell areas as a function of the NaCl concentration for untransfected cells. The boxes represent the first and third quartiles, the whiskers represent the minimum and maximum values, and the red lines represent the mean value. Note that the calculated areas were typical of > 90 % of the transfected and untransfected cells within each field of view, with the remaining cells featuring areas that were larger and/or challenging to estimate accurately. Representative fluorescence images from $n = 3$ biological replicates are shown in (**A**) and (**C**). The cell areas that were calculated for $n = 60$ cells from the $n = 3$ biological replicates are shown in (**B**) and (**D**). A two-sided Student's t test was performed, wherein $P < 0.05$ was considered to be statistically significant. [The same representative images and plots corresponding to transfected cells and untransfected cells exposed to the standard NaCl concentration are also shown in Figure S9.]

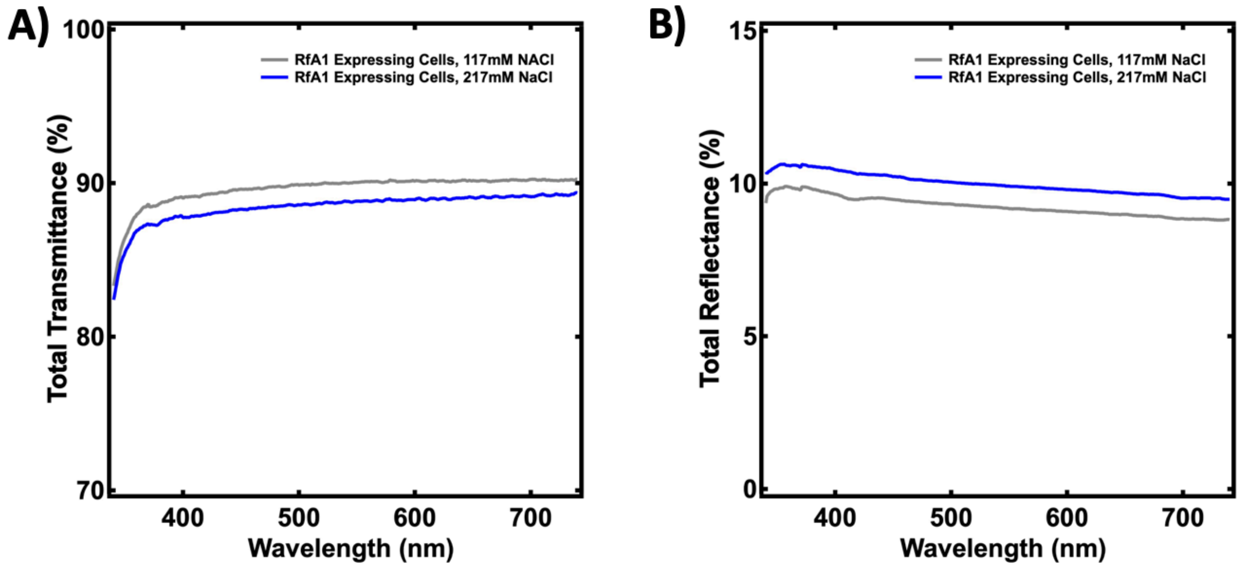

Supplementary Figure 24: Total transmittance and total reflectance of human cells transfected with a vector encoding for the expression of reflectin A1. (**A**) Representative total transmittance spectra obtained for sandwich-type configurations from RfA1-expressing cells after exposure to media with standard, i.e. 117 mM, (gray trace), and higher, i.e. 217 mM, (blue trace) NaCl concentrations. (**B**) Representative total reflectance spectra obtained for sandwich-type configurations from RfA1-expressing cells after exposure to media with standard, i.e. 117 mM, (gray trace) and higher, i.e. 217 mM, (blue trace) NaCl concentrations.

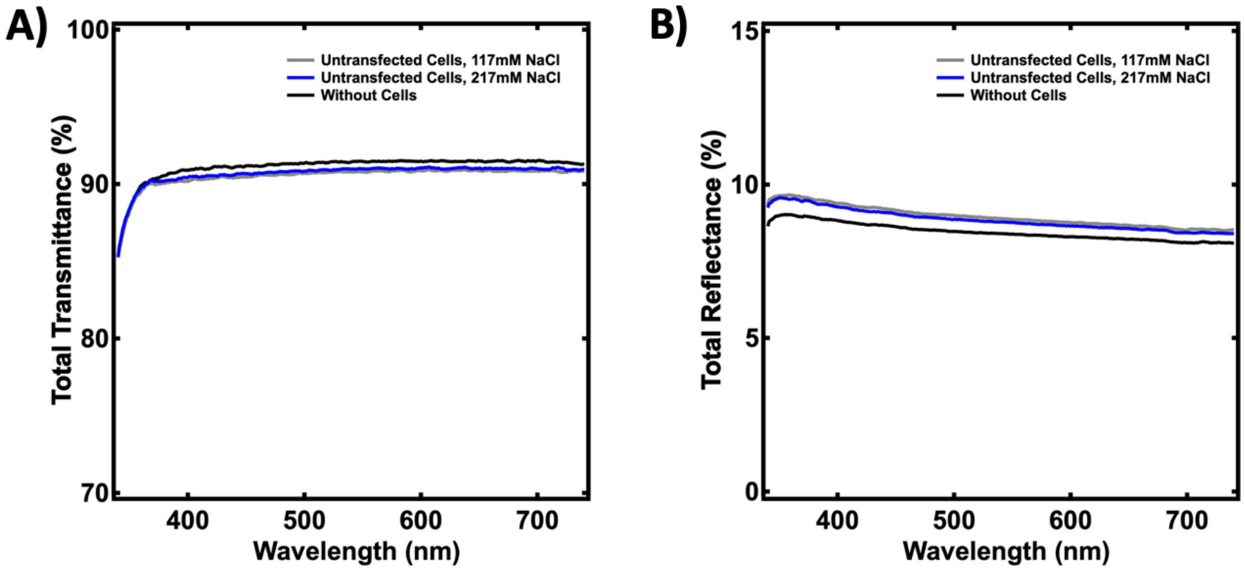

Supplementary Figure 25: Total transmittance and total reflectance of untransfected human cells. (**A**) Representative total transmittance spectra obtained for sandwich-type configurations from untransfected cells after exposure to media with standard, i.e. 117 mM, (gray trace) and higher, i.e. 217 mM, (blue trace) NaCl concentrations. A representative total transmittance spectrum obtained for a sandwich-type configuration in the absence of any cells is shown for comparison (black trace). (**B**) Representative total reflectance spectra obtained for sandwich-type configurations from untransfected cells after exposure to media with standard, i.e. 117 mM, (gray trace) and higher, i.e. 217 mM, (blue trace) NaCl concentrations. A representative total reflectance spectrum obtained for a sandwich-type configuration in the absence of any cells is shown for comparison (black trace).

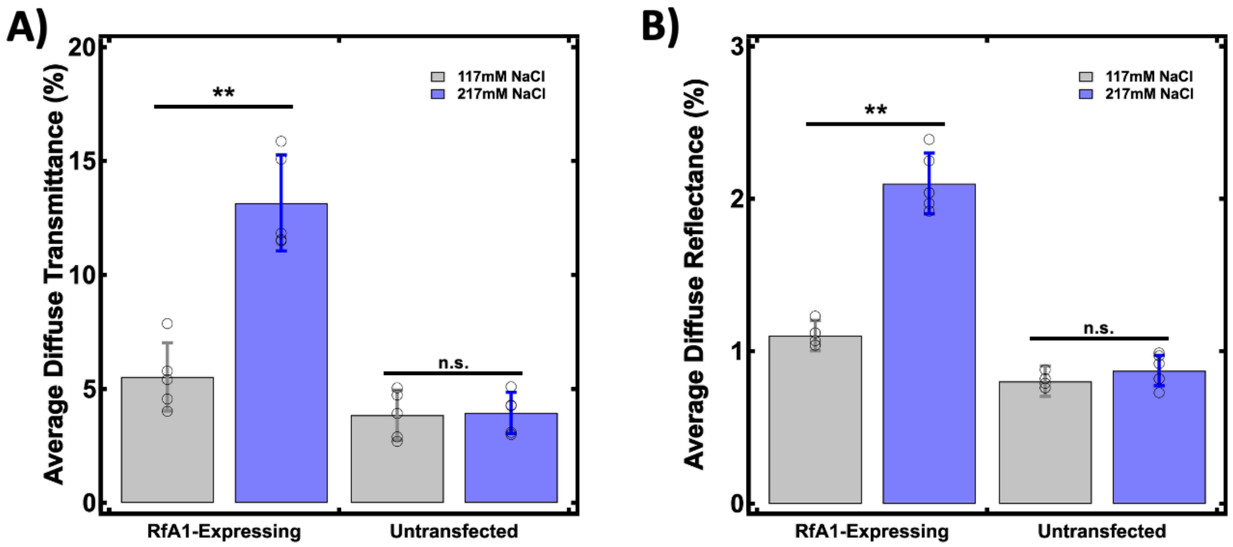

Supplementary Figure 26: Average diffuse transmittance and diffuse reflectance of transfected and untransfected human cells. (**A**) A plot of the average diffuse transmittances for a sandwich-type configuration from RfA1-expressing or untransfected cells after exposure to media with a standard, i.e. 117 mM, (gray bars) or with a higher, i.e. 217 mM, (blue bars) NaCl concentration. The average diffuse transmittances from n = 5 biological replicates are shown, where the bar plots correspond to the mean values and the error bars correspond to the standard deviations. A two-sided Student's t test was performed, wherein $P < 0.05$ was considered to be statistically significant and ** corresponds to $P = 0.0079$. (**B**) A plot of the average diffuse reflectances for a sandwich-type configuration from RfA1-expressing or untransfected cells after exposure to media with a standard, i.e. 117 mM, (gray bars) or with a higher, i.e. 217 mM, (blue bars) NaCl concentration. The average diffuse reflectances from n = 6 biological replicates are shown, where the bar plots correspond to the mean values and the error bars correspond to the standard deviations. A two-sided Student's t test was performed, wherein $P < 0.05$ was considered to be statistically significant and ** corresponds to $P = 0.0022$.

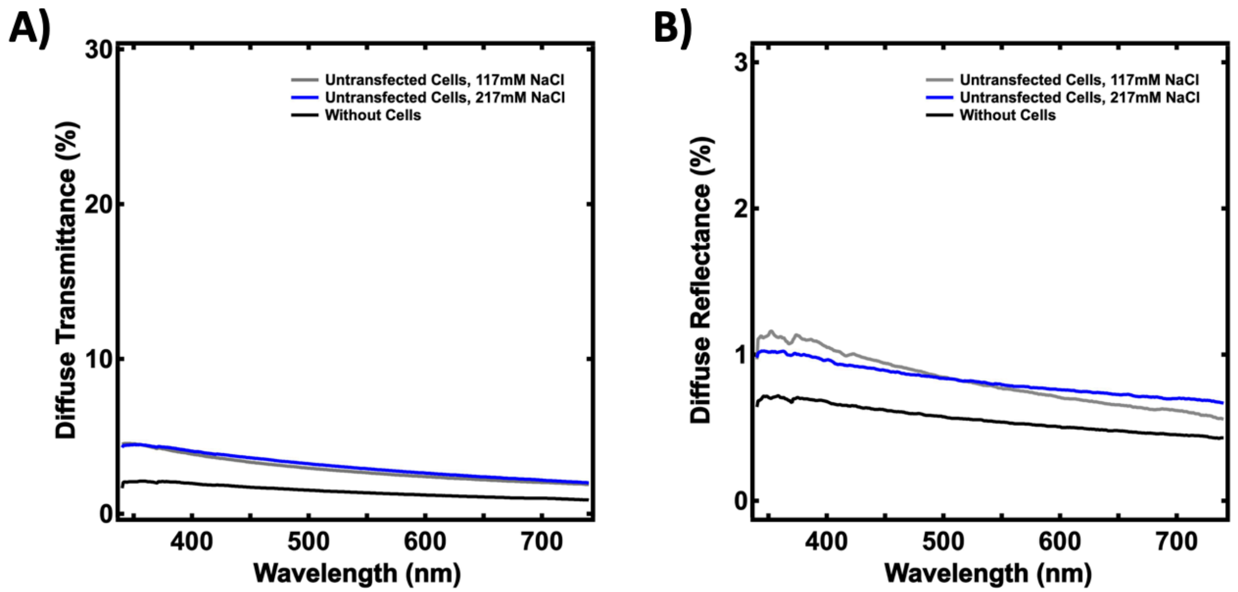

Supplementary Figure 27: Diffuse transmittance and diffuse reflectance of untransfected human cells. (**A**) Representative diffuse transmittance spectra obtained for sandwich-type configurations from untransfected cells after exposure to media with standard, i.e. 117 mM, (gray trace) and higher, i.e. 217 mM, (blue trace) NaCl concentrations. The diffuse transmittance spectrum obtained for a sandwich-type configuration in the absence of any cells is shown for comparison (black trace). (**B**) Representative diffuse reflectance spectra obtained for sandwich-type configurations from untransfected cells after exposure to media with standard, i.e. 117 mM, (gray trace) and higher, i.e. 217 mM, (blue trace) NaCl concentrations. The diffuse reflectance spectrum obtained for a sandwich-type configuration in the absence of any cells is shown for comparison (black trace).

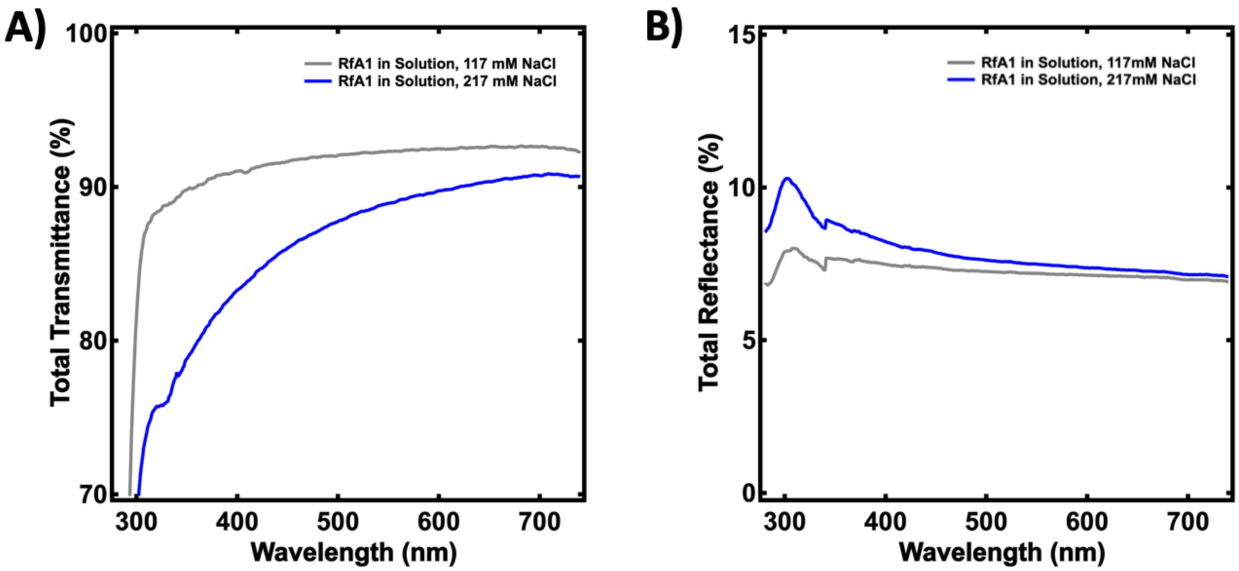

Supplementary Figure 28: Total transmittance and total reflectance of aqueous solutions that contain reflectin A1 nanoparticles. (**A**) Representative total transmittance spectra obtained for aqueous RfA1 solutions with NaCl concentrations of 117 mM (gray trace) and 217 mM (blue trace). (**B**) Representative total reflectance spectra obtained for aqueous RfA1 solutions with NaCl concentrations of 117 mM (gray trace) and 217 mM (blue trace).

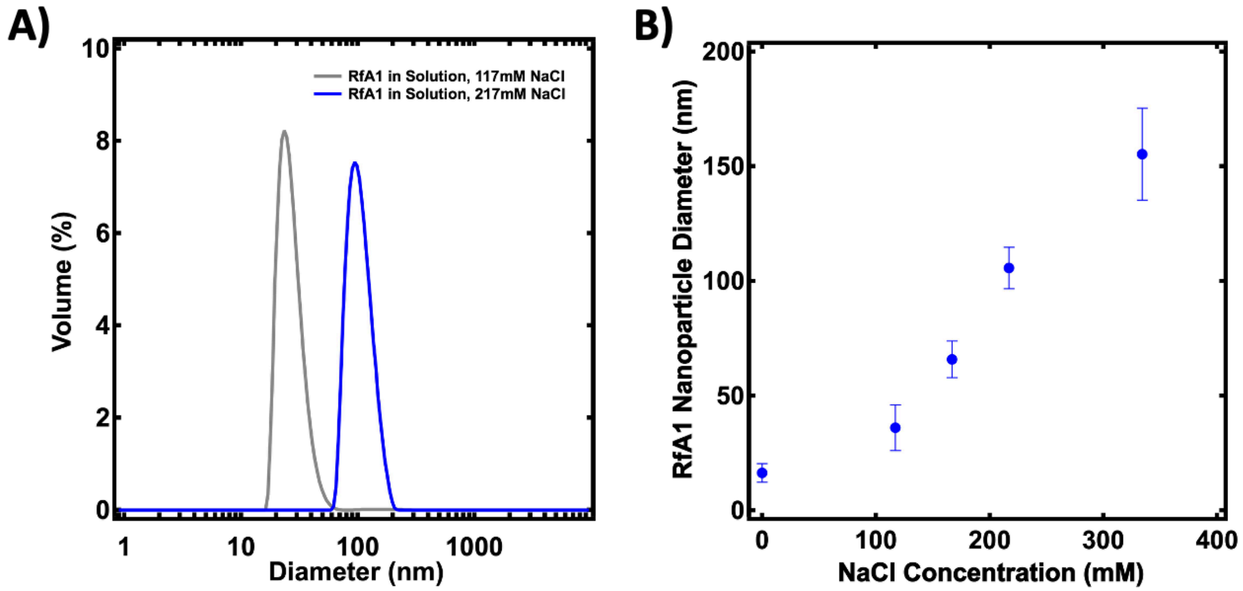

Supplementary Figure 29: Dynamic light scattering of aqueous solutions containing reflectin A1 nanoparticles. (**A**) A representative plot of the volume distribution, which indicates the major particle population, as a function of the nanoparticle diameter for aqueous RfA1 solutions with NaCl concentrations of 117 mM (gray trace) and 217 mM (blue trace). (**B**) A plot of the average nanoparticle diameter for aqueous RfA1 solutions as a function of the NaCl concentration. The nanoparticle diameters from n = 6 biological replicates are shown, where the data points correspond to the mean values and the error bars correspond to the standard deviations.

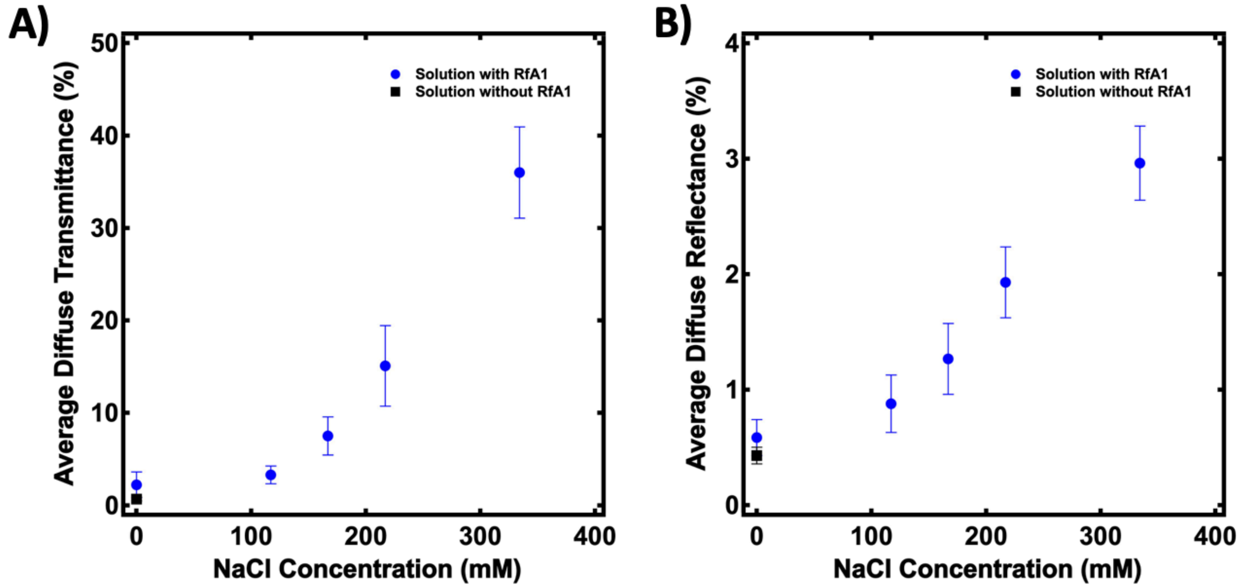

Supplementary Figure 30: Average diffuse transmittance and diffuse reflectance of aqueous solutions containing reflectin A1 nanoparticles as a function of the NaCl concentration. (**A**) A plot of the average diffuse transmittances for aqueous RfA1 solutions as a function of the NaCl concentration (blue circles). The average diffuse transmittance for an aqueous solution lacking any RfA1 nanoparticles (black square) is shown for comparison. (**B**) A plot of the average diffuse reflectances for aqueous RfA1 solutions as a function of the NaCl concentration (blue circles). The average diffuse reflectance for an aqueous solution lacking any RfA1 nanoparticles (black square) is shown for comparison. The average diffuse transmittances and diffuse reflectances from $n = 6$ biological replicates for solutions with RfA1 and from $n = 3$ independent experiments for solutions without RfA1 are shown, where the data points correspond to the mean values and the error bars correspond to the standard deviations.

Supplementary References:

1. DeMartini, D. G. *et al.* Dynamic biophotonics: female squid exhibit sexually dimorphic tunable leucophores and iridocytes. *J. Exp. Biol.* **216**, 3733–3741 (2013).
2. Froesch, D. & Messenger, J. B. On leucophores and the chromatic unit of Octopus vulgaris. *J. Zool. Lond.* **186**, 163–173 (1978).
3. Hanlon, R. T., Mäthger, L. M., Bell, G. R. R., Kuzirian, A. M., & Senft, S. L. White reflection from cuttlefish skin leucophores. *Bioinspir. Biomim.* https://doi.org/10.1088/1748-3190/aaa3a9 (2018).
4. The UniProt Consortium. UniProt: the universal protein knowledgebase. *Nucleic Acids Res.* **45**, D158–D169 (2017).
5. Mäthger L. M., *et al.* Bright white scattering from protein spheres in color changing, flexible cuttlefish skin. *Adv. Funct. Mater.* **23**, 3980–3989 (2013).
