# Supplementary material for: Cephalopod-inspired optical engineering of human cells
Source: Nat Commun. 2020 Jun 2;11:2708. doi: 10.1038/s41467-020-16151-6 (PMC7266819; doi:10.1038/s41467-020-16151-6)
Supplement: Supplementary file 4 — Description of Additional Supplementary Files [file 41467_2020_16151_MOESM4_ESM.pdf]

Supplementary Movie Legends:

Title: Supplementary Movie 1:
Description: Phase video for human cells that express reflectin A1. A time-lapse movie of the changes in phase for human cells containing moving RfA1-based structures, which was obtained ~ 24 h after transfection. The video was generated from phase images that were obtained every minute over a period of 2 hours. The movie has been sped up to 10 frames per second. The white scale bar is 10 μm.

Title: Supplementary Movie 2:
Description: Optical pathlength video for human cells that express reflectin A1. A time-lapse movie of the change in optical pathlength for human cells containing moving RfA1-based structures, which was obtained ~ 24 h after transfection. The video was generated from optical pathlength maps that were calculated for every minute over a period of 2 hours. The movie has been sped up to 10 frames per second. The white scale bar on the bottom (for the cell size) is 10 μm, and the colored scale bar on the right (for optical pathlength) is 325 nm.
