# Supplementary material for: Cephalopod-inspired optical engineering of human cells
Source: Nat Commun. 2020 Jun 2;11:2708. doi: 10.1038/s41467-020-16151-6 (PMC7266819; doi:10.1038/s41467-020-16151-6)
Supplement: Supplementary file 5 — Reporting Summary [file 41467_2020_16151_MOESM5_ESM.pdf]

nature research

Corresponding author(s): Gorodetsky, Alon

Last updated by author(s): Mar. 27, 2020

Reporting Summary

Nature Research wishes to improve the reproducibility of the work that we publish. This form provides structure for consistency and transparency in reporting. For further information on Nature Research policies, see Authors & Referees and the Editorial Policy Checklist .

Statistics

For all statistical analyses, confirm that the following items are present in the figure legend, table legend, main text, or Methods section.

n/a	Confirmed	
☐	☒	The exact sample size (*n*) for each experimental group/condition, given as a discrete number and unit of measurement
☐	☒	A statement on whether measurements were taken from distinct samples or whether the same sample was measured repeatedly
☐	☒	The statistical test(s) used AND whether they are one- or two-sided<br>*Only common tests should be described solely by name; describe more complex techniques in the Methods section.*
☒	☐	A description of all covariates tested
☒	☐	A description of any assumptions or corrections, such as tests of normality and adjustment for multiple comparisons
☐	☒	A full description of the statistical parameters including central tendency (e.g. means) or other basic estimates (e.g. regression coefficient) AND variation (e.g. standard deviation) or associated estimates of uncertainty (e.g. confidence intervals)
☐	☒	For null hypothesis testing, the test statistic (e.g. *F*, *t*, *r*) with confidence intervals, effect sizes, degrees of freedom and *P* value noted<br>*Give P values as exact values whenever suitable.*
☒	☐	For Bayesian analysis, information on the choice of priors and Markov chain Monte Carlo settings
☒	☐	For hierarchical and complex designs, identification of the appropriate level for tests and full reporting of outcomes
☒	☐	Estimates of effect sizes (e.g. Cohen's *d*, Pearson's *r*), indicating how they were calculated

Our web collection on statistics for biologists contains articles on many of the points above.

Software and code

Policy information about availability of computer code

Data collection	The Gene Designer Software (ATUM) was used to design the plasmids used in the study. The ZEN software was used to collect images using the confocal microscope (LSM 780), a MATLAB 2017a GUI was used to collect images from the low-coherence quantitative phase microscope (as described in Yamauchi, T., Iwai, H., Miwa, M., & Yamashita, Y. Opt. Express 16, 12227–12238 (2008)), and the Malvern Panalytical software was used to collect dynamic light scattering measurements from the Zeta- Sizer Nano S.
Data analysis	Data collected in this manuscript were analyzed with: ImageJ (v 2.0.0-rc-69/1.52i) and MATLAB 2017a; data was plotted with Igor Pro 6.1, and statistical analyses were performed with Prism v.8, as specified in the Supplementary Information.

For manuscripts utilizing custom algorithms or software that are central to the research but not yet described in published literature, software must be made available to editors/reviewers. We strongly encourage code deposition in a community repository (e.g. GitHub). See the Nature Research guidelines for submitting code & software for further information.

Data

Policy information about availability of data

All manuscripts must include a data availability statement. This statement should provide the following information, where applicable:

- Accession codes, unique identifiers, or web links for publicly available datasets
- A list of figures that have associated raw data
- A description of any restrictions on data availability

All data needed to evaluate the conclusions in the paper are present in the paper and/or the supplementary materials.

Field-specific reporting

Please select the one below that is the best fit for your research. If you are not sure, read the appropriate sections before making your selection.

☒ Life sciences ☐ Behavioural & social sciences ☐ Ecological, evolutionary & environmental sciences

For a reference copy of the document with all sections, see nature.com/documents/nr-reporting-summary-flat.pdf

Life sciences study design

All studies must disclose on these points even when the disclosure is negative.

Sample size

Representative phase contrast and fluorescence microscopy images from a minimum of 5 independent experiments are shown in the manuscript (Supplementary Figure 7 for the RfA1-expressing cells and for the untransfected cells, and Supplementary Figure 14 for the RfA1 and RFP co-expressing cells). Representative immunofluorescence images from a minimum of 5 independent experiments are shown in the manuscript (Figure 2A and Supplementary Figure 4 for the RfA1-expressing cells, Supplementary Figure 5 for the "mock" transfected cells, Supplementary Figure 6 for the untransfected cells, and Figure 2C and Supplementary Figure 15 for RfA1- and RFP-expressing cells). Representative cross-sectional TEM images from a minimum of 3 independent experiments are shown in the manuscript (Figure 2B and Supplementary Figure 10 for RfA1 expressing cells, Supplementary Figure 12 for untransfected cells, Supplementary Figure 13 for RFP expressing cells, and Supplementary Figure 15 for RfA1- and RFP-expressing cells). Representative RLC-QPM and TLC-QPM images and videos from a minimum of 5 independent experiments are shown in the manuscript (Figure 3A for RLC-QPM of RfA1 expressing cells, Figure 3B and Supplementary Figure 17 for RfA1- and RFP-expressing cells, as well as Figure 3C, Figure 3D, Supplementary Movie 1, and Supplementary Movie 2 for TLC-QPM of RfA1 expressing cells). Representative brightfield microscopy images from a minimum of 5 independent experiments are shown in the manuscript (Figure 4A for RfA1 expressing cells and Supplementary Figure 20 for untransfected cells). Representative total transmittance, total reflectance, diffuse transmittance, and diffuse reflectance spectra from a minimum of 5 independent human cell culture experiments are shown in the manuscript (Supplementary Figure 24 and Figure 4B for RfA1 expressing cells and Supplementary Figures 25 and 27 for untransfected cells). Representative optical characterization data (digital camera images, transmittance and reflectance spectroscopy, and dynamic light scattering) from a minimum of 5 independent reflectin solutions are shown in the manuscript (Figure 4C, Figure 4D, Supplementary Figure 28, Supplementary Figure 29, and Supplementary Figure 30).

A minimum of 3 biologically independent experiment were typically performed to ensure reproducibility of the data as is standard in the literature. To ensure experimental rigour, each result was confirmed via multiple techniques.

Data exclusions

Data were evaluated based on listed quality control criteria (e.g. data were from dishes with cell confluencies of 50-75%). All data that met the quality control criteria were analyzed.

Replication

Our experiments were typically reproduced at least 3 and usually 5 times, to confirm that the data were reproducible. Statistical analysis was performed for all relevant quantifications of data (Figures S9, S22, S23, and S26) to further confirm the trends observed were reproducible.

Randomization

All independent experiments performed used different passages of HEK 293 cells or heterologously expressed protein, as necessary. Thus, all allocations were random.

Blinding

Confocal and Fluorescence Microscopy: All samples were number coded so that during sample imaging, the identity of the sample would not be known to the person imaging, as an additional strategy for removing bias.

Immunofluorescence Microscopy: All samples were number coded so that during sample imaging, the identity of the sample would not be known to the person imaging, as an additional strategy for removing bias.

Transmission Electron Microscopy: All samples were number coded so that during sample imaging, the identity of the sample would not be known to the person imaging, as an additional strategy for removing bias.

Immuno-Electron Microscopy: All samples were number coded so that during sample imaging, the identity of the sample would not be known to the person imaging, as an additional strategy for removing bias.

Reflection Mode Low-Coherence Quantitative Microscopy: Since the fluorescence images were taken separately from the phase images, and only the phase images needed to be analyzed to calculate the optical pathlength and refractive index maps, the analysis was performed without knowledge of which cells were fluorescent (and thus had the protein of interest).

Transmission Mode Low-Coherence Quantitative Microscopy: The quantification of the refractive indices require the local changes in phase as well as the diameter of the region of interest; as such all heights and diameters were notated prior to calculation of refractive indices, to prevent any preference for specific phase differences or diameters.

Brightfield Microscopy: All samples were number coded so that during microscopy, the identity of the sample would not be known, and thus all images would be attained without bias.

Transmittance and Reflectance Spectroscopy: All samples were number coded so that during spectroscopy, the identity of the sample would not be known, and thus all spectra would be attained without bias.

Dynamic Light Scattering: All samples were color coded so that during spectroscopy, the identity of the sample would not be known, and thus

all spectra would be attained without bias.

Reporting for specific materials, systems and methods

We require information from authors about some types of materials, experimental systems and methods used in many studies. Here, indicate whether each material, system or method listed is relevant to your study. If you are not sure if a list item applies to your research, read the appropriate section before selecting a response.

Materials & experimental systems

n/a	Involved in the study
☐	☒ Antibodies
☐	☒ Eukaryotic cell lines
☒	☐ Palaeontology
☒	☐ Animals and other organisms
☒	☐ Human research participants
☒	☐ Clinical data

Methods

n/a	Involved in the study
☒	☐ ChIP-seq
☒	☐ Flow cytometry
☒	☐ MRI-based neuroimaging

Antibodies

Antibodies used

A) Oligoclonal rabbit anti-histidine tag primary antibody (ThermoScientific, 710286)
B) Polyclonal rabbit anti-reflectin primary antibody (Crookes, W. J. et al. Science 303, 235-238 (2004).)
C) Goat anti-rabbit IgG Aleza 488 secondary antibody (ThermoScientific, A-11008)
D) Goat anti-rabbit secondary IgG conjugated to a 12 nm gold nanoparticle (Jackson Immuno Research, 111-205-144)

Validation

A) Oligoclonal rabbit anti-histidine tag primary antibody (ThermoScientific, 710286): Validation from the supplier can be found here: https://www.thermofisher.com/antibody/product/6x-His-Tag-Antibody-clone-21HCLC-Recombinant-Polyclonal/710286; additional validation performed for the data presented in the manuscript is shown in Supplementary Figure 4.

B) Polyclonal rabbit anti-reflectin primary antibody (Crookes, W. J. et al. Science 303, 235-238 (2004).): Validation can be found in the original manuscript describing the first use of the antibody; additional validation performed for the data presented in the manuscript are shown in Supplementary Figures 4, 5, 6, and 15. The antibody was also validated on the heterologously expressed reflectin via Western Blotting (data not shown).

C) Goat anti-rabbit IgG Aleza 488 secondary antibody (ThermoScientific, A-11008):Validation from the supplier can be found here: https://www. thermofisher.com/antibody/product/Goat-anti-Rabbit-IgG-H-L-Cross-Adsorbed-Secondary-Antibody-Polyclonal/A-11008; additional validation performed for the data presented in the manuscript are shown in Supplementary Figures 4, 5, 6, and 15.

D) Goat anti-rabbit secondary IgG conjugated to a 12 nm gold nanoparticle (Jackson Immuno Research, 111-205-144): Validation was performed by checking for the non-specific adsorption of this antibody on reflectin-expressing cell cross-sections, which showed no non-specific adsorption (data not shown).

Eukaryotic cell lines

Policy information about cell lines

Cell line source(s)

ATCC (https://www.atcc.org/products/all/CRL-1573.aspx)

Authentication

Cell lines were not authenticated

Mycoplasma contamination

Cell lines were not tested for mycoplasma contamination

Commonly misidentified lines
(See ICLAC register)

HEK 293 cells were used in the study, and are commonly misidentified with HeLa cells. For the study we separately purchased multiple batches of HEK 293 cells, verified by the supplier to be HEK 293 cells, and there were no HeLa cell cultures within the cell culture facility being used at the time.

HEK 293 cells were chosen for the study because these cells reliably express various recombinat proteins and have been shown to accumulate some highly over-expressed foreign proteins within cytoplasmic inclusion bodies or phase-separated aggregates.
